# Variation in genomic traits of microbial communities among ecosystems

**DOI:** 10.1093/femsmc/xtab020

**Published:** 2021-12-01

**Authors:** Peter F Chuckran, Bruce A Hungate, Egbert Schwartz, Paul Dijkstra

**Affiliations:** Center for Ecosystem Science and Society (ECOSS) and Department of Biological Sciences, Northern Arizona University, Flagstaff, Arizona, United States of America; Center for Ecosystem Science and Society (ECOSS) and Department of Biological Sciences, Northern Arizona University, Flagstaff, Arizona, United States of America; Center for Ecosystem Science and Society (ECOSS) and Department of Biological Sciences, Northern Arizona University, Flagstaff, Arizona, United States of America; Center for Ecosystem Science and Society (ECOSS) and Department of Biological Sciences, Northern Arizona University, Flagstaff, Arizona, United States of America

**Keywords:** genome size, GC content, streamlining, soil, metagenomics, sigma-factors

## Abstract

Free-living bacteria in nutrient limited environments often exhibit traits which may reduce the cost of reproduction, such as smaller genome size, low GC content and fewer sigma (σ) factor and 16S rRNA gene copies. Despite the potential utility of these traits to detect relationships between microbial communities and ecosystem-scale properties, few studies have assessed these traits on a community-scale. Here, we analysed these traits from publicly available metagenomes derived from marine, soil, host-associated and thermophilic communities. In marine and thermophilic communities, genome size and GC content declined in parallel, consistent with genomic streamlining, with GC content in thermophilic communities generally higher than in marine systems. In contrast, soil communities averaging smaller genomes featured higher GC content and were often from low-carbon environments, suggesting unique selection pressures in soil bacteria. The abundance of specific σ-factors varied with average genome size and ecosystem type. In oceans, abundance of *fliA*, a σ-factor controlling flagella biosynthesis, was positively correlated with community average genome size—reflecting known trade-offs between nutrient conservation and chemotaxis. In soils, a high abundance of the stress response σ-factor gene *rpoS* was associated with smaller average genome size and often located in harsh and/or carbon-limited environments—a result which tracks features observed in culture and indicates an increased capacity for stress response in nutrient-poor soils. This work shows how ecosystem-specific constraints are associated with trade-offs which are embedded in the genomic features of bacteria in microbial communities, and which can be detected at the community level, highlighting the importance of genomic features in microbial community analysis.

## Teaser

Distribution of genomic traits in microbial communities between ecosystems.

## INTRODUCTION

Assessing microbial communities through a trait-based framework highlights important relationships between microbes and their environment which may not be detectable through taxonomic analyses alone (Green, Bohannan and Whitaker 2008; Raes *et al*. 2011; Barberán *et al*. 2014; Fierer, Barberán and Laughlin 2014; Krause *et al*. 2014; Martiny *et al*. 2015). Notably, genomic characteristics such as genome size, GC content, number of regulatory genes and number of 16S rRNA gene copies, have been shown to be indicators for growth rates (Vieira-Silva and Rocha 2010), life-history strategies (Cobo-Simón and Tamames 2017) and population dynamics (Batut *et al*. 2014) of bacteria. Relationships between genomic features and environmental factors such as nutrient usage (Batut *et al*. 2014; Giovannoni, Cameron Thrash and Temperton 2014; Roller, Stoddard and Schmidt 2016), aboveground cover (Schmidt *et al*. 2018; Li *et al*. 2019), temperature (Sabath *et al*. 2013) and precipitation (Gravuer and Eskelinen 2017) have additionally demonstrated the potential utility of genomic traits for assessing the relationship between bacteria and their environment.

The genome size of free-living bacteria may be reduced by a process called genomic streamlining, wherein nutrient limitation selects for smaller genomes as a way to reduce the cost of reproduction (Giovannoni *et al*. 2005). Streamlined genomes are associated with a number of traits which also reduce reproductive costs, most notably a lower GC content (which reduces nitrogen requirements and is less costly to synthesize), fewer regulatory genes (specifically those encoding σ-factors), smaller intergenic spacer regions, and fewer 16S rRNA gene copies (Giovannoni, Cameron Thrash and Temperton 2014). Consequently, bacteria with streamlined genomes are thought to have a higher resource use efficiency and lower maximum growth rates compared to bacteria with larger genomes and more rRNA gene copies (Lauro *et al*. 2009), although evidence for this relationship remains mixed (Klappenbach, Dunbar and Schmidt 2000; Vieira-Silva and Rocha 2010; Yooseph *et al*. 2010; Karcagi *et al*. 2016; Kirchman 2016; Kurokawa *et al*. 2016). Streamlining has long-been known to be highly prevalent in marine systems (Morris *et al*. 2002) where the streamlined SAR11 clade, with a genome of only ∼1.3 Mbp, makes up 25% of all planktonic bacteria (Giovannoni 2017). As a result, much of the current knowledge regarding streamlining is based on marine systems. However, the recently described streamlined (2.81 Mbp) Verrucomicrobia, *Candidatus Udaeobacter copiosus*, has been shown to be ubiquitous in soils, comprising up to 30% of recovered taxa in some grassland soils (Brewer *et al*. 2017)—indicating that genome reduction may also be an important force shaping soil bacteria.

Temperature can also influence genome size due to increased fitness of small cells at high temperatures (Sabath *et al*. 2013). Accordingly, small cells and smaller genomes are typically associated with higher optimal growth temperatures. This relationship is most pronounced in thermophilic communities (Wang, Cen and Zhao 2015), but has also been demonstrated in marine systems (Swan *et al*. 2013; Morán *et al*. 2015; Huete-Stauffer *et al*. 2016) and more recently in soils (Sorensen *et al*. 2019). These patterns between genome size, GC content and number of 16S rRNA gene copies as a result of temperature-induced genome reduction often resemble patterns in streamlined genomes (Sabath *et al*. 2013).

Small genomes are also prevalent in host-associated bacteria. However, the processes underpinning the reduction in genome size involve several mechanisms, including drift, rapid mutation rate or other mechanisms, which could be more important than streamlining (Batut *et al*. 2014). In environments where nutrients are abundant but population sizes small, deletions in bacterial genomes are more likely to become fixed in a population (Mira, Ochman and Moran 2001; Batut *et al*. 2014), a process particularly common in host-associated gut microbiota, where population sizes are small due to isolation (McCutcheon and Moran 2012). Bacteria subject to higher levels of mutation are more likely to be AT-rich since there is a mutational bias from GC → AT (Kuo, Moran and Ochman 2009; Hershberg and Petrov 2010; Hildebrand, Meyer and Eyre-Walker 2010; Batut *et al*. 2014). Since the mechanisms driving the evolution of host-associated bacteria often stray from streamlining, genome reduction in host-associated bacteria may yield different patterns in genome reduction. Specifically, streamlining, which is more a directional rather than stochastic process, will often select for specific genes (Batut *et al*. 2014).

Much of this knowledge concerning bacterial genomic traits has been derived from cultures or isolates. This presents substantial bias in our understanding of these relationships (Gweon, Bailey and Read 2017), especially for genomic traits of bacteria in complex microbial communities (Rinke *et al*. 2013), as most bacterial taxa have never been cultured or isolated. An alternative approach is to examine genomic traits on a community level *in situ*. By observing community-derived metrics of genomic traits we broaden our understanding of the distribution and implication of these traits as they occur in the natural world. This is an important practice for microbial ecology as there has been growing interest in trait dimensions which might improve our assessment of community function (analogous to those existing for plants; Westoby *et al*. 2021), yet little work has been done to observe these traits on the community level. Such metrics could be valuable in the comparison of communities across landscapes and ecosystems. Genomic traits such as GC content, number of regulatory genes and average genome size may be especially useful for this purpose, as they can often be easily estimated from metagenomic datasets and do not require an extensive knowledge of the taxa within the community. The relative ease with which these traits may be derived makes them ideal metrics for large-scale comparisons and represents a potentially valuable tool for linking microbial communities with ecosystem-level processes.

The ability to leverage these traits to gain insight into function, assembly or evolutionary relationships remains untested. A necessary step towards building a more comprehensive understanding of community-derived traits includes assessment of the distribution of these traits across systems, such has been done numerous times for isolates. Here, we present a comparison of genomic traits from 116 metagenomes from soil, marine, host-associated and thermophilic systems. These systems were chosen as they represent distinct environments which exert unique evolutionary pressures on genomic traits which might produce predictable outcomes: streamlining in oceans; temperature-induced genome reduction in thermophiles and drift in host-associated communities. Several mechanisms have been shown to influence genome size in soils; however, the predominant force is not well-understood. Isolate genomes in soils tend to be comparatively larger than other systems (Sabath *et al*. 2013), which is thought to be a result of the increased metabolic diversity (Barberán *et al*. 2014). The overall aim of this study is to assess whether genomic traits measured at the community level track relationships which have been observed in isolates. Accordingly, we hypothesize that, consistent with trends in isolates, the average genome size in soil microbial communities will be larger than in marine, host-associated or thermophilic communities. We also predict that GC content will be positively correlated with average genome size in free-living soil, marine and thermophilic communities—consistent with trends from streamlined and thermophilic isolates. Finally, we predict that while both free-living and host-associated communities with small average genome sizes will demonstrate a low GC content, free-living communities will also exhibit additional streamlined traits such as a reduced number of σ-factor and rRNA gene copies.

## MATERIALS AND METHODS

### Dataset curation

Metagenomes from soil, marine, thermophilic and host-associated communities were downloaded from the Integrated Microbial Genomes and Microbiomes (IMG/M; Chen *et al*. 2019) system. Data were used in accordance to JGI IMG/M data release policies (https://jgi.doe.gov/user-programs/pmo-overview/policies/), and studies were only used under the follow conditions: (1) The studies were previously published with a corresponding publication on the IMG database or; (2) We were granted written consent from the team which generated the data. This publication does not act as a primary publication for these studies and use of the data from the second group requires consent from the corresponding principal investigators of that study. We searched for soil and marine samples that were untreated and collected *in situ* systems (i.e. not an incubation or microcosm). If studies included any form of experimental manipulation, then only metagenomes from the control were selected. For thermophilic samples we searched for communities derived from natural hot-springs, and for host-associated samples we focused on animal-associated communities. We then selected samples which were both sequenced and assembled (MEGAHIT; Li *et al*. 2015 or SPAdes; Bankevich *et al*. 2012) by the Joint Genome Institute (JGI) and where > 35 Mbp were assembled. Replicates appearing to be derived from a single sample (i.e. identical metadata and sample name) were discarded. In order to limit potential bias introduced by a specific study site or set of protocols of a given study, no more than four samples were used from any single geographical location and no more than 14 samples were selected from a single study. Ecosystem type was determined for soil samples using the available metadata and study description. In total, 116 samples from 30 different studies were used in this analysis (Figure S1 and Tables S1 and S2, Supporting Information; Baker *et al*. 2015; Rossmassler *et al*. 2015; Cardenas *et al*. 2015, 2018; Leung *et al*. 2016; Ouyang 2016; Whitman *et al*. 2016; Beam *et al*. 2016; Wilhelm *et al*. 2017a,b,c; Gravuer and Eskelinen 2017; Hawley *et al*. 2017; Armstrong *et al*. 2018; Lee *et al*. 2018; Maresca *et al*. 2018; Colatriano *et al*. 2018; Krüger *et al*. 2019; Camargo *et al*. 2019; Abraham *et al*. 2020; Hervé *et al*. 2020; Mushinski *et al*. 2020; Nayfach *et al*. 2020; Ouyang and Norton 2020; Li *et al*. 2021; Williams *et al*. 2021).

Average genome size for each metagenome was estimated using the program MicrobeCensus (parameters -n 50 000 000; Nayfach and Pollard 2015) on QC filtered reads accessed through the JGI Genome Portal (Nordberg *et al*. 2014). MicrobeCensus uses the abundance of single-copy genes to estimate the number of individuals in a population, which is then divided by the total number of read base-pairs to provide an estimate of the average genome size in a metagenome.

From IMG/M, we accessed the size of the metagenomic sample (bp), GC-%, total number of 16S rRNA gene copies and the total number of σ factors identified by the KEGG Orthology database (Table 1; KEGG–Kanehisa and Goto 2000). We estimated the number of genomes per metagenome by dividing the total base pair count of the metagenome by the estimated average genome size from MicrobeCensus. The average number of 16S rRNA gene copies per genome and the number of σ-factors gene copies per genome was then determined by dividing the total number of 16S rRNA or σ-factor gene copies by the estimated number of genomes.

**Table 1:** Gene name, description and KEGG ortholog identifier (K numbers) for each σ-factor used in the analysis.

σ-factor gene	Functions regulated by σ-factor	K Number
***rpoD***	Primary sigma factor, "Housekeeping" (Lonetto, Gribskov and Gross 1992)	KO:K03086
***rpoE***	Envelope stress (Hayden and Ades 2008)	KO:K03088
***fliA***	Flagella biosynthesis (Ohnishi *et al*. 1990)	KO:K02405
***rpoH***	Heat shock (Grossman, Erickson and Gross 1984)	KO:K03089
***sigI***	Heat shock (Zuber, Drzewiecki and Hecker 2001)	KO:K03093
***sigH***	Heat shock, oxidative stress (Fernandes *et al*. 1999)	KO:K03091
***rpoN***	Nitrogen assimilation (Ronson *et al*. 1987; Totten, Cano Lara and Lory 1990), motility (Totten, Cano Lara and Lory 1990) and quorum sensing (Heurlier *et al*. 2003)	KO:K03092
***rpoS***	Stress response (Battesti, Majdalani and Gottesman 2011; Hengge 2014) and stationary phase (Lange and Hengge-Aronis 1991)	KO:K03087
***sigB***	Stress response (Hecker, Schumann and Völker 1996) and stationary phase (Boylan, Redfield and Price 1993)	KO:K03090

To ensure that any observed trends were not heavily influenced by the abundance of nonbacterial genomes, such as large eukaryotic genomes, we assessed the relationship between average genome size and the relative abundance of assembled bacterial reads. For each metagenome, we accessed the taxonomic assignments of mapped reads from IMG/M and then summed the total number of reads grouped by domain. The relationship between the relative abundance of bacteria and average genome size of the community was then calculated for each ecosystem to assign a cutoff which demonstrated the least amount of bias (as determined by linear regression). As a result, samples where bacteria made up less than < 95% of the assembled reads were discarded.

Since archaeal abundance in thermophilic microbial communities is often high, filtering samples with < 95% bacterial reads discarded a large number of thermophilic samples. Post-filtering, only five thermophilic samples were left for analysis—a sample size ultimately too small to generate conclusions. Rather than omitting the thermophilic environments from our analysis entirely, and because small archaeal genomes abundance have been shown to be correlated with higher optimum growth temperatures (Sabath *et al*. 2013), we decided to include thermophilic samples with > 5% archaeal abundance in several of the comparisons. Although these data do not examine bacterial streamlining specifically, we find that they still provide valuable insight into how genomic traits are distributed in these communities. Mixed thermophilic samples (those including > 5% archaea) are shown separately in figures and analyses. In comparisons of genome size versus bacteria-specific traits, such as 16S rRNA gene copies or abundance of sigma factors, we only report samples where bacteria comprise > 95% of annotated reads.

### Analysis

Multiple regression was used to determine the relationship between genome size and genomic characteristics—specifically, GC content, 16S rRNA gene relative abundance, the relative abundance of the total number of σ-factor genes and the relative abundance of specific σ-factor genes as listed in Table 1. Models were constructed with the command lm or lmer from the R (v3.6.1 (Team 2018)) package lme4 (Bates *et al*. 2020). For each response variable, we constructed multiple models considering all parameters and interactions. Final models were selected using Akaike information criterion (AIC) values. The addition of a new parameter resulting in a reduction of the AIC value by at least 4 indicated a significantly better fit with increased model complexity.

To assess the abundance of σ-factor genes between different ecosystems, we used both the multi-response permutation procedure (MRPP) as well as the permutational multivariate analysis of variance (PERMANOVA). The MRPP was conducted using all samples while PERMANOVA was conducted using 11 randomly selected genomes from each ecosystem to ensure balanced design. Both analyses were conducted using Bray–Curtis dissimilarity matrices constructed from the relative abundance of each σ-factor. To visualize differences in the distribution of different types σ-factors between ecosystems we used nonmetric multidimensional scaling (NMDS) on Bray–Curtis distances. MRPP, PERMANOVA and NMDS were done using the *vegan* package (Oksanen *et al*. 2019) in R (v3.6.1).

### Isolates

To compare relationships between genomic characteristics of a microbial community with characteristics of isolates, we accessed over 6000 isolates of bacteria, archaea and fungi from the IMG/M system in June of 2020. Isolates were selected if they were (1) publicly available; (2) previously published and (3) sequenced by JGI. The associated publications for these isolates may be found in the Supplemental references. Metadata was used to group samples into one of three ecosystem types: soil, marine, thermophilic or host-associated. To avoid potential bias introduced by large studies selecting for specific taxa, we randomly selected no more than 20 isolates from a single study. Relationships between genomic characteristics were analysed using multiple regression analyses as described above for the analysis of community-level traits. ANOVA was used to assess differences in the distribution of genomic characteristics between isolates and metagenomic averages.

## RESULTS

### Average Genome Size and GC Content

**Figure 1.**
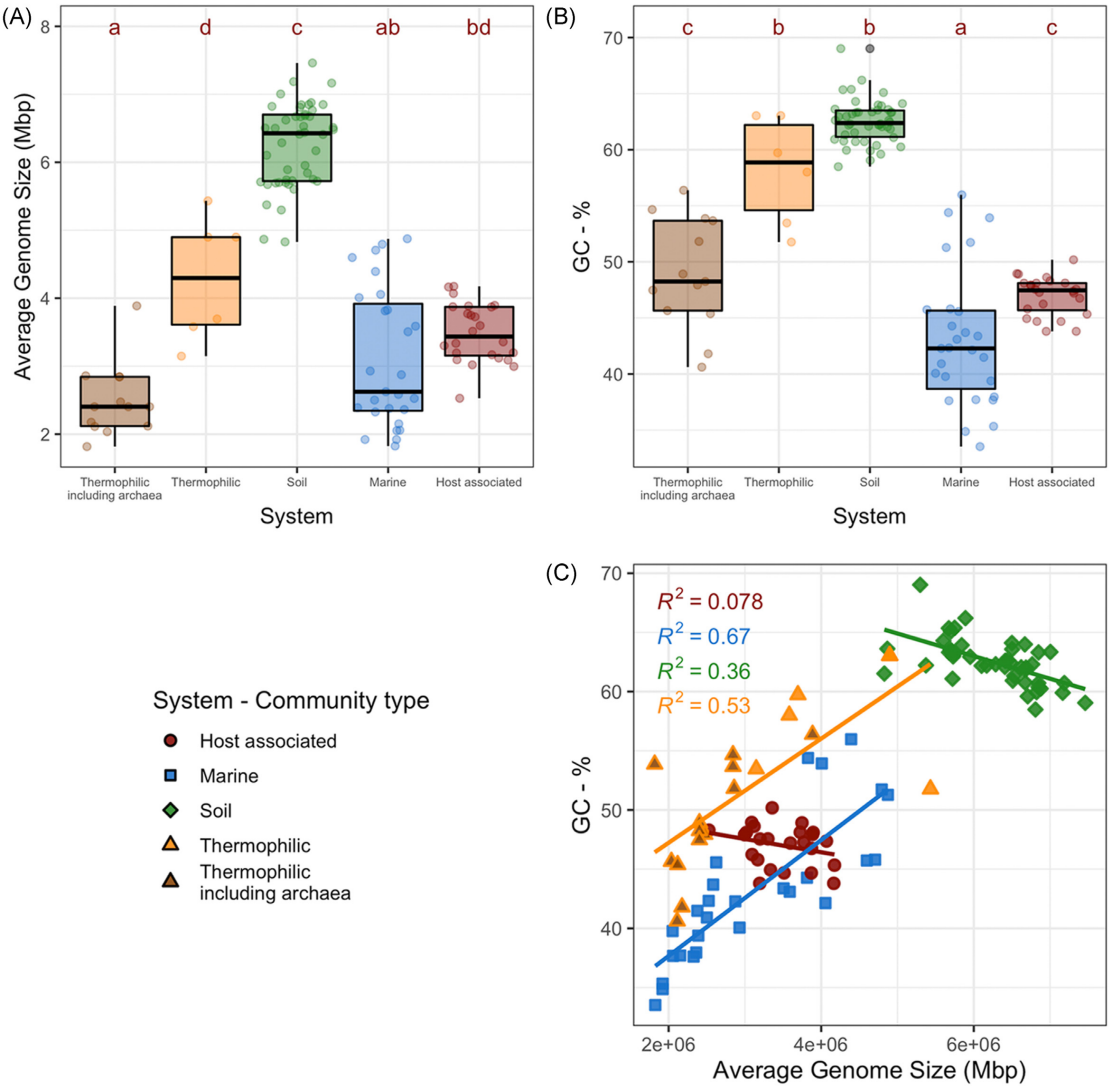
Average genome size and GC-content calculated from environmental metagenomes. **(A)** Boxplots of the average genome size (Mbp) of microbial communities in different ecosystems. **(B)** Boxplots showing GC-% between systems. **(C)** GC-% as a function of average genome size (Mbp) of a metagenome, separated by system. Point shape and outline represent source system; point fill represents system including thermophilic samples with archaea.

**Figure 2.**
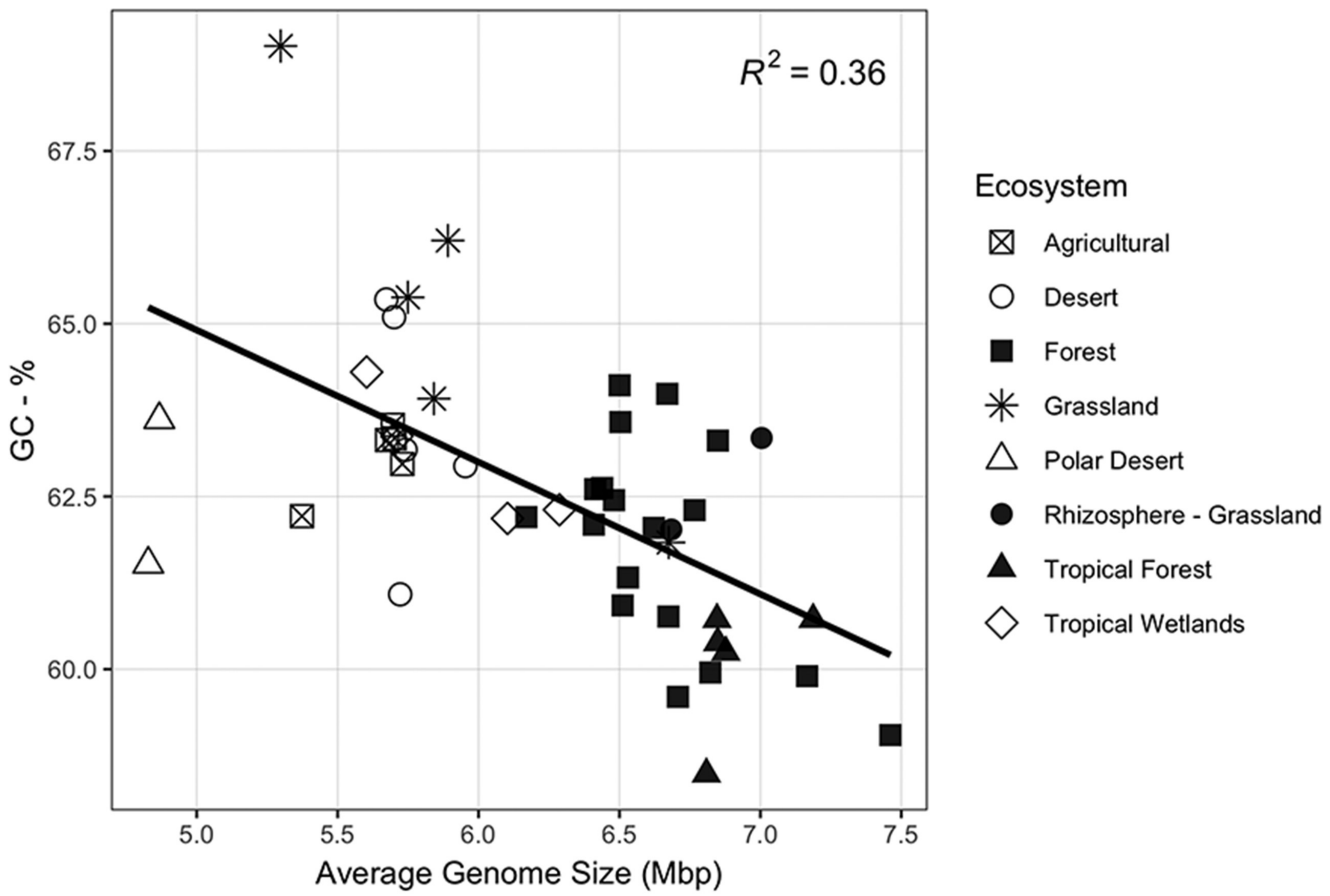
GC content (%) as a function of average genome size (Mbp) in soils, with color indicating source environment.

Average genome size was significantly different between ecosystems (ANOVA; F_4,111_ = 135.9, *P* < 0.01). Specifically, average genome size was higher in soils compared to marine, host-associated, or thermophilic communities (Fig. 1A, Tukey's HSD *P* < 0.01). GC content (%) varied between each ecosystem (ANOVA; F_4,111_ = 140.3, *P* < 0.01), and was highest in soil, followed by thermophilic, host-associated and then marine communities (Fig. 1B). The relationship between GC content and average genome size varied between ecosystems (Fig. 1C). A comparison of multiple models, using AIC values as selection criteria, indicated that GC content was best predicted by average genome size, ecosystem and their interaction (F_9,106_ = 136.1, *P* < 0.01; Table S3, Supporting Information). Specifically, GC content was positively correlated with average genome size in marine and thermophilic communities, negatively correlated in soil communities and not significantly related in host-associated communities (Fig. 1C). The relationship between average genome size and GC content was offset between marine and thermophilic communities, wherein thermophilic communities had a higher GC content than marine communities with the same average genome size (Fig. 1C). The relationship between GC content and average genome size was strongly driven by the abundance of archaea in the mixed thermophilic samples (Figure S2, Supporting Information). In soils, average genome size and GC content were significantly different between ecosystem types (ex. Deserts, grasslands and forests; ANOVA: Mbp—F_7,38_ = 24.35, *P* < 0.01; GC-%—F_7,38_ = 4.986, *P* < 0.01; Fig. 2).

**Figure 3.**
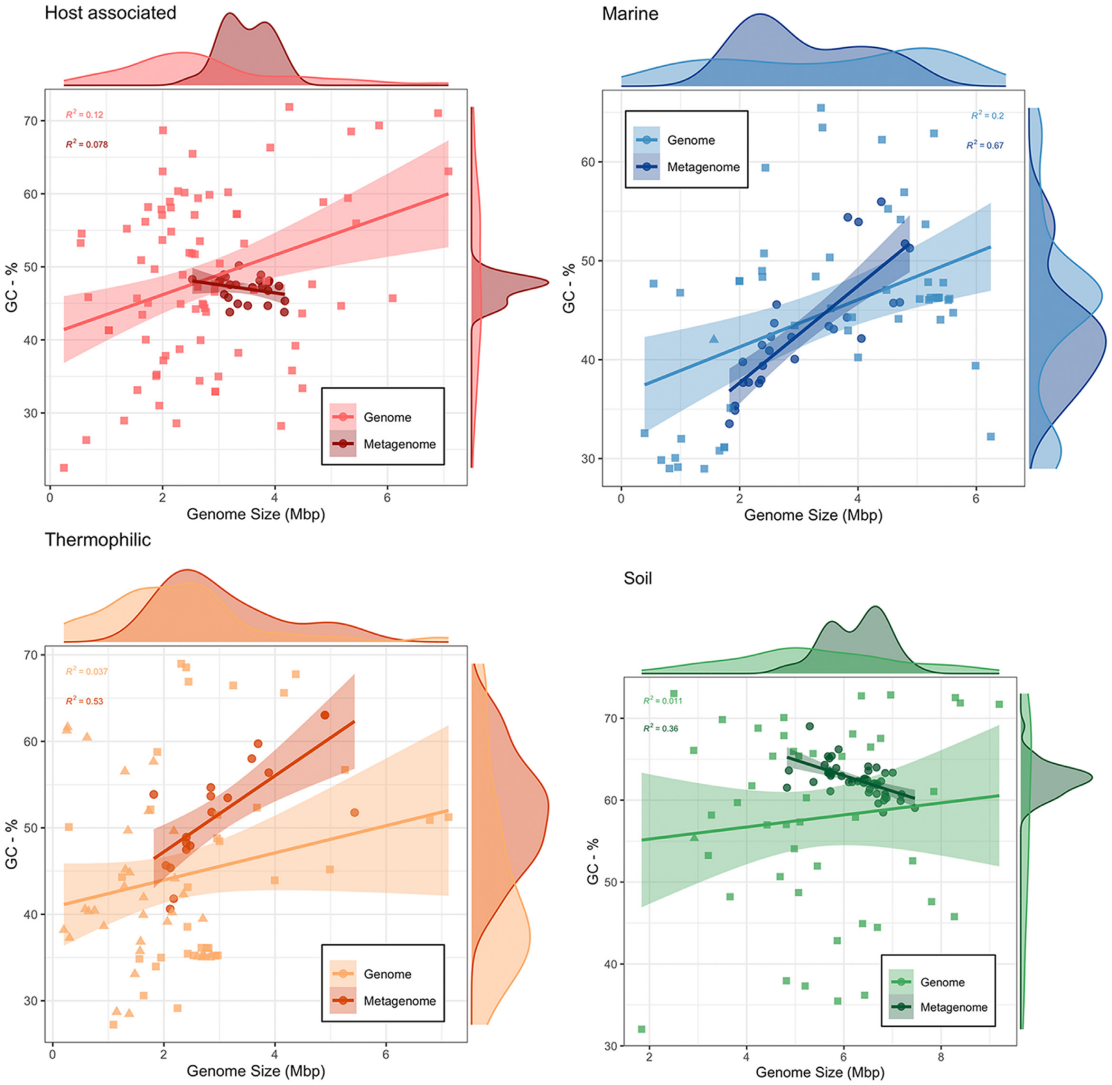
The relationship and distribution of genome size and GC content for isolates and metagenomic averages for each system. In each panel, metagenomes (dark circles) are plotted against bacterial (light squares) and archaeal (light triangles) isolates. Regression lines between genome size and GC-% are shown for both metagenomes (dark lines) and isolates (light lines). Marginal density plots show the distributions of GC-% (right) and genome size (top) for isolates (light) and metagenomic averages (dark).

The average genome size and GC content of the metagenomes fell within the range of isolates from each ecosystem (Fig. 3). However, the mean genome size and GC content derived from metagenomes varied from isolates in both soil and thermophilic environments (ANOVA; *P* < 0.05), but not in marine environments.

### 16S rRNA gene copies and Sigma factors

Host-associated communities had the highest number of 16S rRNA gene copies per genome, followed by soils and then thermophilic and marine communities (Figure S3, Supporting Information). A comparison of AIC values indicated that ecosystem type alone was the best predictor of 16S rRNA gene copies per genome (Figure S3 and Table S3, Supporting Information).

**Figure 4.**
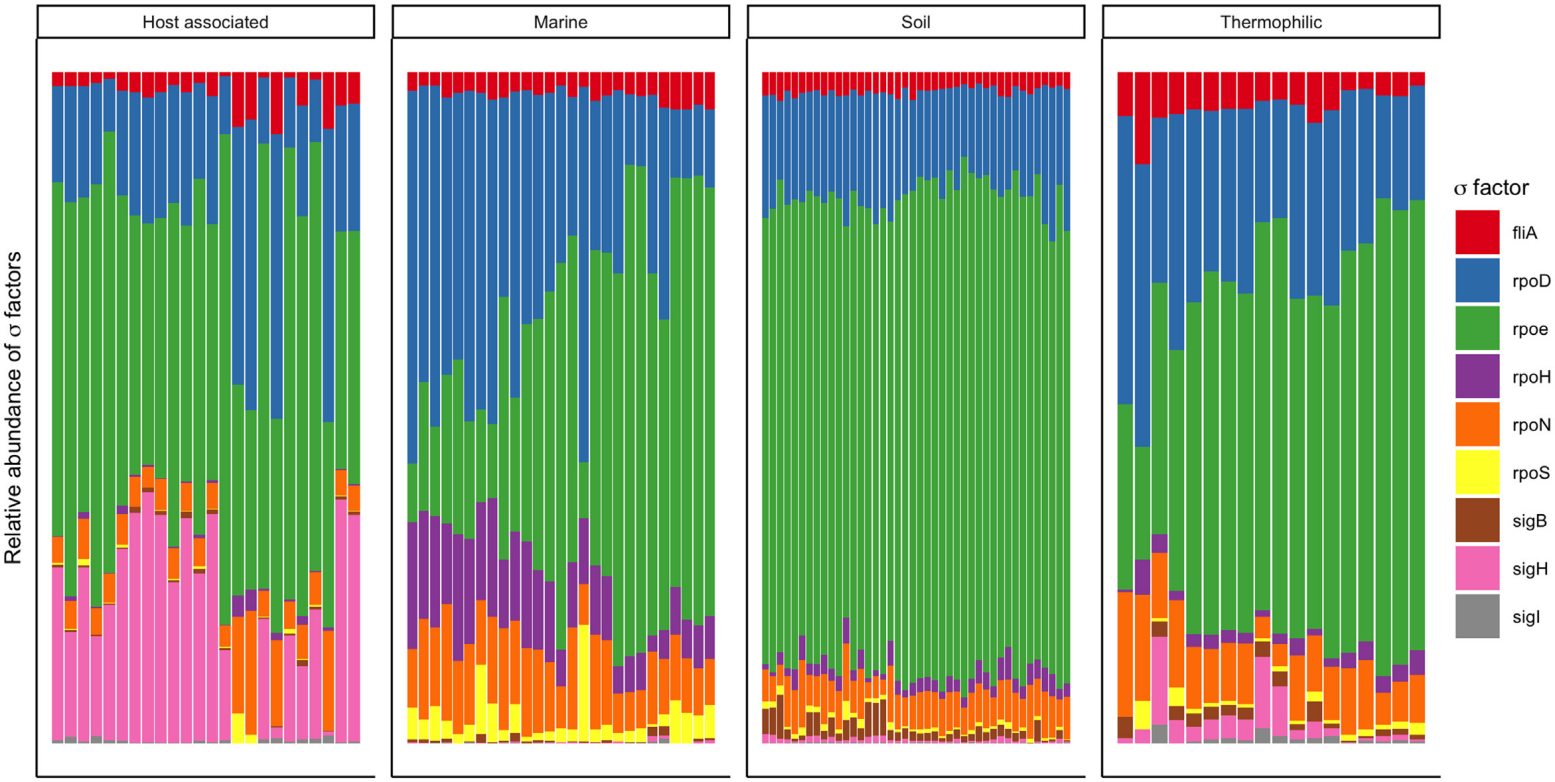
The relative abundance σ-factors in a metagenome separated by ecosystem. Each bar represents the abundance of σ-factors in a single metagenome, and metagenomes are ordered from smallest to largest average genome size (left to right) for each ecosystem.

**Figure 5.**
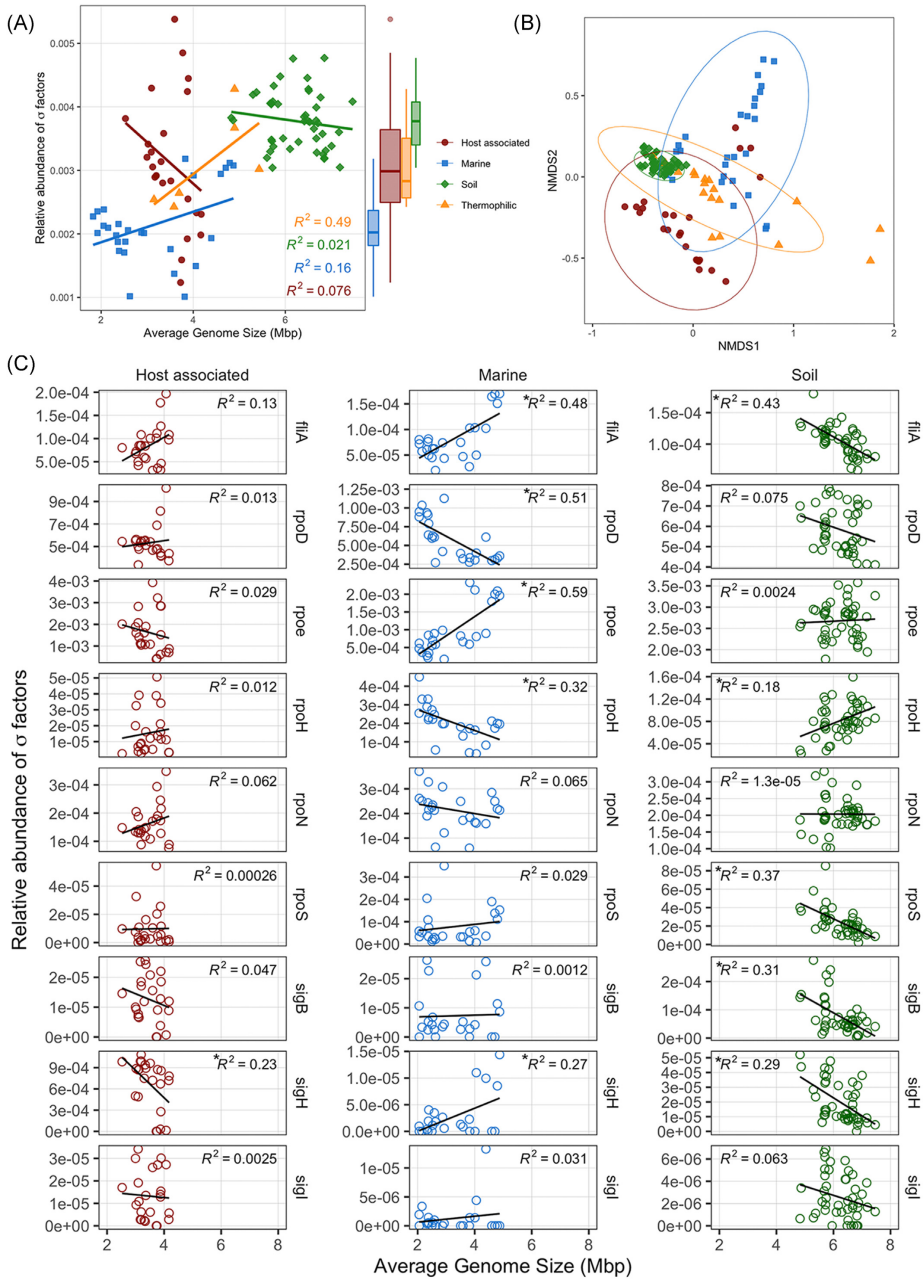
The relative abundance of σ-factors (σ-factor count/total gene count) as a function of average genome size and system. **(A)** The relative abundance of all σ-factors (σ-factor count/total gene count) in a metagenome against average genome size. Source environment indicated by color for host associated (red), soil (green), thermophilic (orange) and marine (blue) communities. **(B)** NMDS of Bray–Curtis distance of the relative abundance of σ-factors (σ-factor count/total gene count) from a metagenome. **(C)** The relative abundance (σ-factor count/total gene count) of 9 σ-factors (rows) versus average genome size, separated by environment (columns). Statistical significance of a relationship (*P* < 0.05) is indicated with an asterisk before *R^2^* value.

The relative abundance of σ-factors genes per metagenome changed with estimates of average genome size and this relationship varied significantly between ecosystems (Figs 4 and 5A; Table S3, Supporting Information). Average genome size was significantly correlated with the relative abundance of σ-factors in thermophilic environments (*R*^2^ = 0.49), but not in soil, marine or host associated environments (*R*^2^ < 0.2; Fig. 5A). The distribution of σ-factor types within a metagenome varied more between ecosystems than within (Figs 4 and 5B; MMRP, A = 0.34, *P* < 0.01), and ecosystems differed significantly (Figs 4 and 5B; PERMANOVA, *R*^2^ = 0.50, *P* < 0.01).

The relationship between average genome size and the relative abundance of individual σ-factors was dependent on both ecosystem type and the type of σ-factor (Fig. 5C; Table S4, Supporting Information). In host-associated communities, the relative abundance of only one σ-factor, *sigH*, was significantly (*P* = 0.018) negatively correlated with average genome size. Abundance of all other sigma factors were unchanged with genome size in host-associated communities (Table S4, Supporting Information). In soil communities the relative abundance of *rpoH* per metagenome significantly increased (*P* < 0.01) with larger average genome size, while the relative abundance per metagenome of *rpoS, sigH, sigB* and *fliA* decreased (*P* < 0.01). In marine communities, we found that the relative abundance of *fliA*, *rpoE* and *sigH* significantly increased (*P* < 0.01) with genome size, and the abundance of *rpoH*, and *rpoD* significantly decreased (*P* < 0.01). Due to the small samples size of thermophilic communities, we did not include the relationships between σ-factors and average genome size for thermophilic environments; however, correlation coefficients and statistics for all linear regressions between average genome size and σ-factor abundance for each ecosystem can be found in Table S4 (Supporting Information). A visualization of average σ-factor copies per genome can be found in Figure S4 (Supporting Information).

## DISCUSSION

The range of values for both genome size and GC content on the community level was substantially more narrow than those recorded for isolates, both from the literature (Sabath *et al*. 2013) and isolates gathered from the IMG database (Fig. 3). However, we did observe considerable variation both between and within different ecosystems. The observed within-ecosystem variation is likely a product of the range of ecosystems included in the analysis. For example, soil metagenomes were derived from deserts, grasslands, forests, tropical forests and polar deserts, and traits accordingly tended to separate out by these habitats (Fig. 2). This work demonstrates the variability that exists within a specific ecosystem type and highlights the potential utility of genomic traits in studies comparing multiple habitat types. Between ecosystems, microbial communities in marine, host and thermophilic environments had a smaller average genome size and lower GC content than those in soil, consistent with our first hypothesis based on previous findings from studies using bacterial isolates and single-amplified genomes (Raes *et al*. 2007; Giovannoni, Cameron Thrash and Temperton 2014; Cobo-Simón and Tamames 2017). Although small genomes may persist in soil communities, larger genomes tend to be more abundant (Barberán *et al*. 2014; Brewer *et al*. 2017); a feature often attributed to the advantage gained from the increased abundance of secondary metabolite genes in large soil genomes (Konstantinidis and Tiedje 2004).

Since smaller genomes tend to have lower GC content (Bentley and Parkhill 2004), we expected to find a positive correlation between GC content and average genome size for each ecosystem. Contrary to our second hypothesis, we only found this relationship in marine and thermophilic communities. This relationship in marine communities is not especially surprising considering how many studies have observed the trade-off between the genome size and GC content of individuals in marine systems. However, our results demonstrate that these trade-offs are detectable on a community scale and emphasizes the degree to which streamlining shapes community-averaged traits. In thermophilic communities, this relationship appeared confounded with the presence of archaea (Figure S2, Supporting Information), thus making it impossible to distinguish between archaeal abundance or temperature as a driver for smaller genome size in these extreme environments. Additionally, higher temperatures might similarly result in smaller archaeal genomes (Sabath *et al*. 2013), further contributing to this signal. It is worth noting that the relationship between genome size and GC content in thermophilic communities was offset higher from marine systems, even for bacterial dominated thermophilic communities. This offset is perhaps the result of a requirement for thermal stability in hot environments which is provided by the GC triple-hydrogen bonds versus the AT double-bond (Wada and Suyama 1986; Musto *et al*. 2006).

Both GC content and average genome size in host-associated communities were low, a common feature of symbiotic bacteria (McCutcheon and Moran 2012). Although host-associated bacteria in small populations often have AT-rich genomes (Batut *et al*. 2014), the relationship between GC content and average genome size was not significant for host-associated communities. Reduced genetic flow in these communities could mean that changes in nucleotide frequency and genome size develop independently in populations. Therefore, these trends might exist within, but not between, communities. In other words, host-associated environments might produce small AT-rich genomes, but these two traits do not covary between communities as in marine systems.

Soil communities exhibited a negative relationship between average genome size and GC content. This does not necessarily exclude streamlining as a driver of genome size in soils but suggests additional drivers of genome size and GC content. One explanation of this relationship is that soil microbial communities skew towards smaller genomes with a higher GC content due to carbon limitation. A GC base pair has a carbon to nitrogen ratio of 9:8 while an AT base pair has a ratio of 10:7. A reduction in GC content, therefore, decreases the amount nitrogen required for DNA synthesis, which has been suggested as an explanation of the low GC content in small genomes that is commonly exhibited in marine systems, where nitrogen is often limiting (Grzymski and Dussaq 2012). In contrast, carbon is generally considered to be the limiting factor for growth in soil bacteria (Demoling, Figueroa and Bååth 2007; Hobbie and Hobbie 2013). A higher GC content might therefore be advantageous when carbon is particularly limiting. This would explain the negative correlation between genome size and GC content in soils—as smaller nutrient-limited soil bacteria would gain a stochiometric advantage from GC rich DNA. In this dataset, communities from deserts, agricultural fields and grasslands had a smaller average genome size and higher GC content (Fig. 2). These environments tend to have lower soil and microbial carbon to nitrogen ratios than forests (Xu, Thornton and Post 2013). Similarly, bacterial communities in forests tended to have larger average genome sizes and lower GC content. Although this mechanism for nucleotide selection has not been established in soils, selection for high GC content in response to carbon limitation is not unfounded (Hellweger, Huang and Luo 2018; Shenhav and Zeevi 2020). Moreover, microbial communities in bare soil have been shown to have a higher GC content than in vegetated soil (Chen *et al*. 2021), and larger genomes were associated with lower GC content in a recent pangenomic study (Choudoir *et al*. 2021). It is important to note that many other environmental factors may fall along the environment gradient shown here, several of which might also influence GC content; such as temperature and moisture, which have been shown to influence nucleotide composition in terrestrial plants (Šmarda *et al*. 2014) and the genomic traits of microbes (Gravuer and Eskelinen 2017; Sorensen *et al*. 2019). Still, our data demonstrate a relationship between genomic traits in soil which is distinct to those of other systems and emphasizes the need to develop a more complete understanding of genomic features across soil microbial communities. A more thorough understanding of these relationships in soil might enhance our ability to use community-derived genomic traits in ecosystem science; for instance, in tracking growth, nutrient turnover and microbial contributions to soil organic carbon on an ecosystem-scale.

Another explanation is that fungal reads may reduce the overall GC content of a metagenome while raising estimates of average genome size. Although we attempted to avoid the influence of fungal genomes by limiting our dataset to metagenomes dominated by bacteria, and found that the abundance of eukaryotic reads to only slightly coincide with the relationship between average genome size (*R*^2^ = 0.12) and GC content (*R*^2^ = 0.14), it still is possible that even a low abundance of large fungal genomes affected our estimates. To assess this further, we applied a more stringent cut-off on the number of eukaryotic assigned reads (<1% of total) which resulted in no detectable relationship between the number or eukaryotic reads and average genome size and GC content (Figure S5a and b, Supporting Information) and found that the relationship between average genome size and GC content stayed intact (Figure S5c, Supporting Information).

Inconsistent with our third hypothesis, we did not find that the relative abundance of σ-factors was associated with average genome size in free-living communities. However, we did observe that marine communities maintained a lower abundance of σ-factor gene copies in comparison to other ecosystems, even when average genome size was comparable. One explanation is that the reduction of σ-factor gene copies is particularly effective in reducing reproductive costs in marine systems. Marine systems are considered to be nutrient poor relative to soils and a general reduction in the proportion of σ-factors in bacterial genomes may function as an adaptation to nutrient constraints. We also found many trends between average genome size and the abundance of specific σ-factor genes in marine communities. In marine metagenomes, the relative abundance per genome of *rpoD* and *rpoH*, which encode for σ^D^ and σ^H^ respectively, was negatively correlated with average genome size. These trends are perhaps caused by the abundance of the streamlined SAR11 clade, which only contain σ^D^ and σ^H^ (Giovannoni 2017). Conversely, the abundance of the gene *fliA*, which encodes for the σ^28^ and regulates flagella biosynthesis (Ohnishi *et al*. 1990), increased with average genome size. This relationship reflects that found in marine systems, wherein nutrient scarcity selects for smaller, more streamlined, cells while increased nutrient availability selects for larger cells capable of chemotaxis (Lauro *et al*. 2009; Stocker 2012).

In soils, the relative abundance of many σ-factors were negatively correlated with estimates of average genome size. Most notably, we observed a decrease in the relative abundance of *rpoS* (σ^S^) but no significant change in the abundance of *rpoD* (σ^D^) with increasing average genome size. The balance between *rpoS* and *rpoD* may be a trade-off between stress tolerance and growth (Ferenci 2003; Nyström 2004). A higher ratio of *rpoS* to *rpoD* has been shown to increase the cell's capacity to cope with stress but limit its ability to grow on a variety of carbon sources (Ferenci 2003; King *et al*. 2004; Maharjan *et al*. 2013). We see this reflected in the environments from which the metagenomes were samples, with microbial communities from high stress environments, such as deserts, having a higher abundance or *rpoS* compared to lower-stress carbon-rich environments, such as forests (Figure S6, Supporting Information).

Surprisingly, we found a high abundance of *fliA* gene copies in soil communities with smaller genomes, several of which were sourced from desert environments. Motility may be more valuable in nutrient limited soil environments, whereas in environments with high nutrient inputs, nutritional competency may be more paramount. However, these results contrast with the commonly held notion that chemotaxis is most prevalent in mesic soils. One explanation is that motility may be especially important when water availability is ephemeral. A greater number of regulatory mechanisms would, therefore, be advantageous as it would allow for a rapid response to periodic pulses of moisture. Another possibility is that bacteria utilize biofilms surrounding fungal hyphae, or ‘fungal highways’ (Kohlmeier *et al*. 2005), which could explain the persistence of flagellated bacteria even in xeric environments (Pion *et al*. 2013).

Finally, we found that the distribution of genomic traits estimated from soil and hot-spring communities did not follow the distribution derived from isolates—potentially due to a decoupling of traits between the individual and community level. The relationship between genome size and GC content was also substantially different between soil isolates and isolates of soil bacteria. These results indicate that certain ecosystem trade-offs may be detectable using community-derived estimates of microbial traits as opposed to isolates and showcases how relating these traits to specific environments may reveal important ecosystem-level pressures on microbial community traits.

However, it is necessary to consider that the data used for this comparison were not sourced from the same studies and the sample size was fairly limited. If genomic traits are to be used as trait-dimensions in microbial ecology, more work must be done observing the distribution of these traits both within and between communities. Further, we found that many of the studies we were able to access were collected from more specialized communities. Although we believe that the comparison of these communities still has merit in showing the range of genomic traits for particular systems, they might not accurately reflect the true distribution of these traits in their respective environments globally.

## CONCLUSION

We found several compelling ecosystem-specific relationships between genomic traits of a microbial community, most notably with genome size, GC content and the distribution of σ-factors. Several of these relationships align with evolutionary mechanisms which relate to known drivers in these environments, such as streamlining in oceans and drift in host-associated communities. We also observed trends in soils which were not in-line with known mechanisms of genome reduction, emphasizing the need to develop an understanding of the controls of genomic features in soils. In this way, our work demonstrates the importance of genomic traits in the field of microbial ecology and ecosystem science; both in their potential to assess microbial communities via ecosystem-specific trade-offs, as well as their ability to reveal new selection pressures not detectable through the analysis of individuals.

## DATA AVAILABILITY

Studies were used with permission from the principal investigators and according to JGI data release policies (https://jgi.doe.gov/user-programs/pmo-overview/policies/). A full list of studies included in this publication can be found in Table S1. This publication does not act as a primary publication for these data, nor do associated publications represent that the corresponding study is publicly available.

## Supplementary Material

xtab020_Supplemental_Files
