# Supplementary material for: Variation in genomic traits of microbial communities among ecosystems
Source: FEMS Microbes. 2021 Dec 1;2:xtab020. doi: 10.1093/femsmc/xtab020 (PMC10117780; doi:10.1093/femsmc/xtab020)

Supplemental Table 1:

Study name, GOLD Study ID and relevant publications of studies used in the analysis. This publication does not act as a primary publication for these data, nor do associated publications represent that the corresponding study is publicly available. Studies were used with permission from the principal investigators and according to JGI data release policies (https://jgi.doe.gov/user-programs/pmo-overview/policies/).

GOLD Study ID	Study Name	Associated Publications or permission acquired
**Gs0114436**	Agricultural soil microbial communities from Utah and Georgia to study Nitrogen management	Ouyang 2016, 2020
**Gs0127393**	Amended soil microbial communities from New York, USA to study carbon cycling	Whitmann 2016
**Gs0120398**	Animal gut microbial communities from fecal samples from Wisconsin, USA	Written permission
**Gs0114433**	Aqueous microbial communities from the Delaware River/Bay and Chesapeake Bay under freshwater to marine salinity gradient to study organic matter cycling in a time-series	Maresca 2018
**Gs0111485**	Bacterial and archaeal communities from various locations to study Microbial Dark Matter (Phase II)	Written permission
**Gs0131207**	Coastal seawater microbial communities from Marineland, Florida, United States (2017 CSP-EMSL call)	Written permission
**Gs0128773**	Comprehensive metagenome and single cell genome sequencing from the open ocean community of North Pacfic Subtropical Gyre, Station ALOHA	Written permission
**Gs0084161**	Cubitermes and Nasutitermes termite gut microbial communities from Max Planck Institute for Terrestrial Microbiology, Germany	Herve 2020; Rossmassler2015
**Gs0071004**	Forest soil microbial communities from multiple locations in Canada and USA	Wilhelm 2017abc; Cardenas 2015, 2018; Mohn 2017
**Gs0135153**	Hardwood forest soil microbial communities from various locations in the United States	Mushinski 2020
**Gs0095507**	Hot spring microbial communities from Yellowstone National Park	Lee 2018
**Gs0095504**	Marine gutless worms symbiont microbial communities from various locations	Written permission
**Gs0046785**	Marine microbial communities from expanding oxygen minimum zones in the northeastern subarctic Pacific Ocean	Written permission
**Gs0127371**	Marine microbial communities from the Costa Rica Dome and surrounding waters	Written permission

**Gs0110176**	Marine microbial communities from the Southern Atlantic Ocean affecting the dissolved organic carbon pool	Written permission
**Gs0114292**	Marine microbial communities from the Southern Atlantic ocean transect to study dissolved organic matter and carbon cycling	Written permission
**Gs0127566**	Methane metabolizing microbial communities from different methane-rich environments from various locations	Collection described in Armstrong 2018
**Gs0110190**	Microbial and viral regulation of community carbon cycling across diverse low-oxygen zones	Written permission
**Gs0084160**	Pelagic marine microbial communities from North Sea	Krüger 2018
**Gs0131210**	Plant-associated microbial communities from Velloziaceae species in rupestrian grasslands, the National Park of Serra do Cipo, Brazil	Camargo 2019
**Gs0053075**	Polar desert microbial communities from Antarctic Dry Valleys	Written permission
**Gs0114820**	Saline lake microbial communities from various lakes in Antarctica	Williams 2021
**Gs0067861**	Saline, thermophilic phototrophic and chemotrophic mat microbial communities from various locations in USA and Mexico	Written permission
**Gs0120349**	Serpentine soil microbial communities from UC McLaughlin Reserve, CA, USA	Gravuer 2017
**Gs0136050**	Soil and marine airborne microbial communities from various locations	Written permission
**Gs0136122**	Soil microbial communities from Loxahatchee National Wildlife Refuge, Florida, United States	Abraham 2020
**Gs0133409**	Soil microbial communities from Purdue University Martell Research Forest, Indiana, United States	Written permission
**Gs0128792**	Systems level insights into methane cycling in arid and semi-arid ecosystems	Written permission
**Gs0121362**	Terrestrial soil microbial communities with and without Nitrogen fertilizer from Kellogg Biological Station, Michigan, USA	Li 2021
**Gs0075432**	Tropical forest soil microbial communities from Luquillo Experimental Forest, Puerto Rico	Written permission

Supplemental Table 2:

Sample name, IMG reference number, and associated study for all metagenomes used in analysis. Please see file **`Supplemental_Table_2.csv`.** This publication does not act as a primary publication for these data and subsequent use of these samples should only be used according to the JGI data release policies (https://jgi.doe.gov/user-programs/pmo-overview/policies/).

Supplemental Table 3:

Comparison of models predicting GC-%, 16s rRNA gene copy relative abundance, and sigma-factor relative abundance. Model comparisons were conducted using AIC values as selection criteria. New parameters which reduced the AIC value by > 4 were added to the model. The optimum model for each response variable is highlighted in bold.

Response	Model	df	AIC
GC-%	System	4	645.4616
	Genome Size	3	701.8270
	Genome Size + System	5	628.2078
	**Genome Size x System**	**11**	**570.8312**
Estimated 16s rRNA genes copies per genome			
	Null	2	570.1399
	**System**	**5**	**518.8544**
	Genome Size (Mbp)	3	563.2023
	Genome Size (Mbp) + System	6	516.8588
	Genome Size (Mbp) x System	9	511.3246
Sigma-factors (relative abundance)			
	Genome Size (Mbp)	3	-1183.713
	**System**	**5**	**-1206.336**
	Genome Size (Mbp) + System	6	-1205.114
	Genome Size (Mbp) x System	9	-1210.136

Supplemental Table 4:

Output of linear regression models determining the relationship between genome size and the relative abundance of sigma factors for each ecosystem type. Coefficients and statistics highlighted in bold represent strongly significant relationships (P < 0.01). Bolded and italicized output represents weakly significant relationships (0.05 > P > 0.01).

	Host associated				Marine			
	B	$F_{1,22}$	R-sq	p-value	B	$F_{1,25}$	R-sq	p-value
fliA	3.46E-05	3.26	0.09	8.48E-02	**2.95E-05**	**25.14**	**0.48**	**3.60E-05**
rpoD	3.46E-05	0.28	-0.03	6.00E-01	**-2.41E-04**	**35.32**	**0.57**	**3.33E-06**
rpoE	-3.70E-04	0.65	-0.16	4.30E-01	**5.32E-04**	**43.10**	**0.62**	**7.06E-07**
rpoH	3.47E-06	0.26	-0.03	6.13E-01	**-7.02E-05**	**19.15**	**0.41**	**1.88E-04**
rpoN	3.68E-05	1.45	0.02	2.40E-01	-2.16E-05	2.50	0.05	1.26E-01
rpoS	4.77E-07	0.01	-0.05	9.40E-01	8.83E-06	0.34	-0.03	5.63E-01
sigB	-3.93E-06	1.09	0.00	3.09E-01	-8.06E-08	0.00	-0.04	9.59E-01
sigH	***-0.0003879***	***6.39***	***0.19***	***1.92E-02***	**1.89E-06**	**8.13**	**0.22**	**8.61E-03**
sigI	-1.25E-06	0.05	-0.04	9.18E-01	5.35E-07	1.09	0.00	3.07E-01

	Soil				Thermophilic			
	B	$F_{1,44}$	R-sq	p-value	B	$F_{1,4}$	R-sq	p-value
fliA	**-2.51E-05**	**33.26**	**0.43**	**7.41E-07**	-2.66E-05	1.26	0.04	3.25E-01
rpoD	-4.87E-05	3.57	0.07	6.55E-02	-4.77E-05	3.74	0.35	1.25E-01
rpoE	3.38E-05	0.11	0.00	7.45E-01	6.10E-04	6.24	0.51	6.68E-02
rpoH	**2.02E-05**	**9.58**	**0.18**	**3.41E-03**	**3.91E-05**	**52.7**	**0.91**	**1.91E-03**
rpoN	-2.77E-07	0.00	0.00	9.81E-01	-3.22E-06	0.06	-0.23	8.16E-01
rpoS	**-1.44E-05**	**25.30**	**0.37**	**8.73E-06**	1.05E-05	2.97	0.28	1.59E-01
sigB	**-5.63E-05**	**19.36**	**0.31**	**6.79E-05**	-7.47E-06	0.33	-0.15	5.29E-01
sigH	**-1.22E-05**	**18.31**	**0.29**	**9.97E-05**	-1.04E-05	0.99	-0.01	3.75E-01
sigI	-8.15E-07	2.95	0.06	9.29E-02	-3.01E-06	0.43	-0.13	5.49E-01

Supplemental Figure 1:

Map showing the central location of each study. Study system is indicated by color and symbol shape.

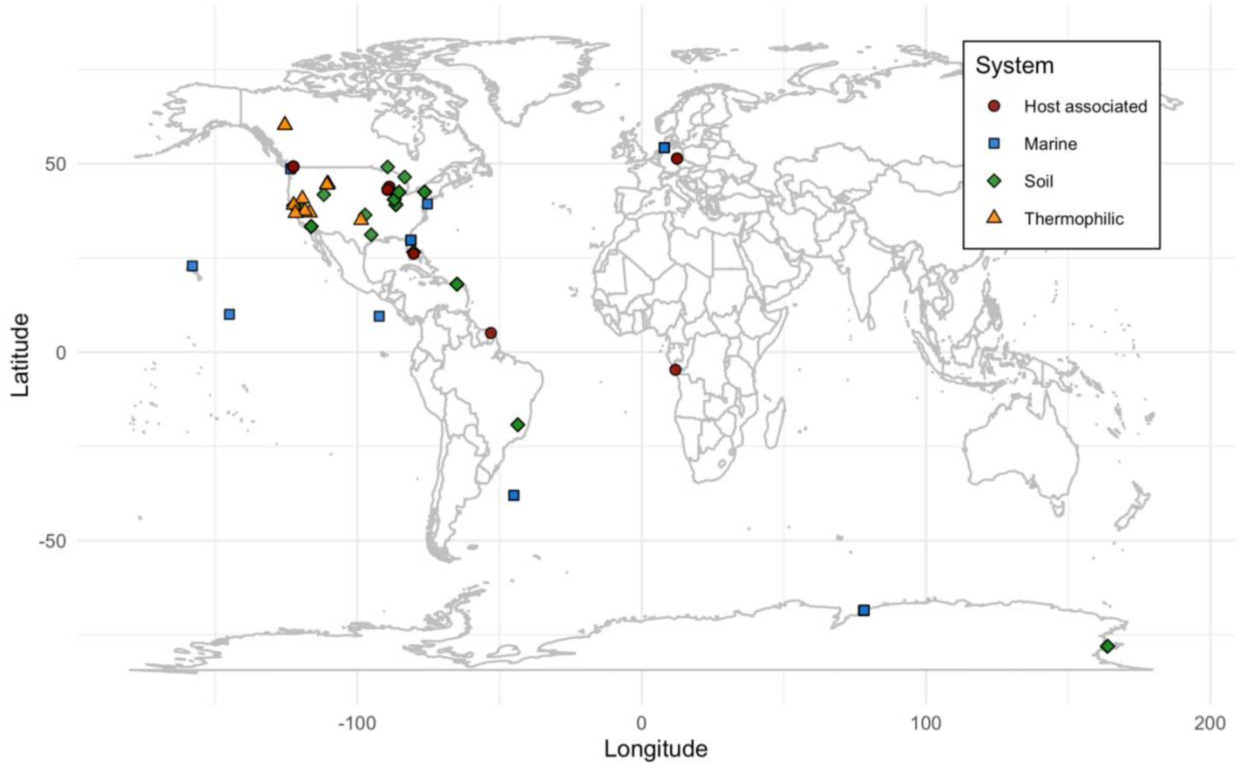

Supplemental Figure 2:

For thermophilic communities, GC content (%; **A**) and average genome size (Mbp; **B**) as a function of archaeal relative abundance (% of annotated reads).

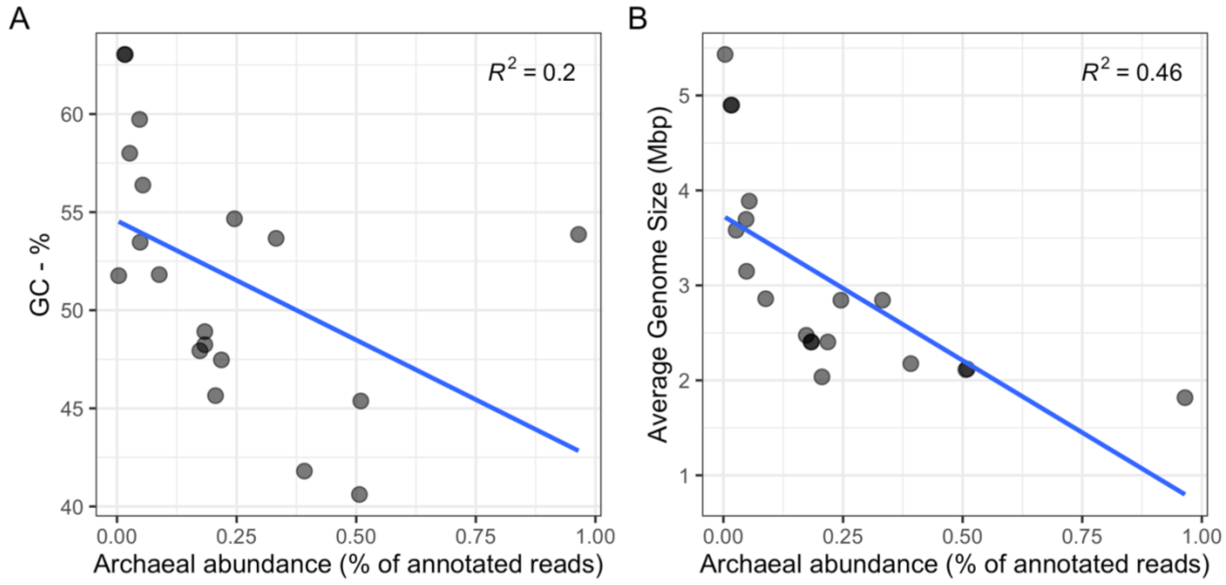

Supplemental Figure 3:

Estimated 16s copies per genome between ecosystems (**A**), and by average genome size with slopes shown separately for each ecosystem (**B**).

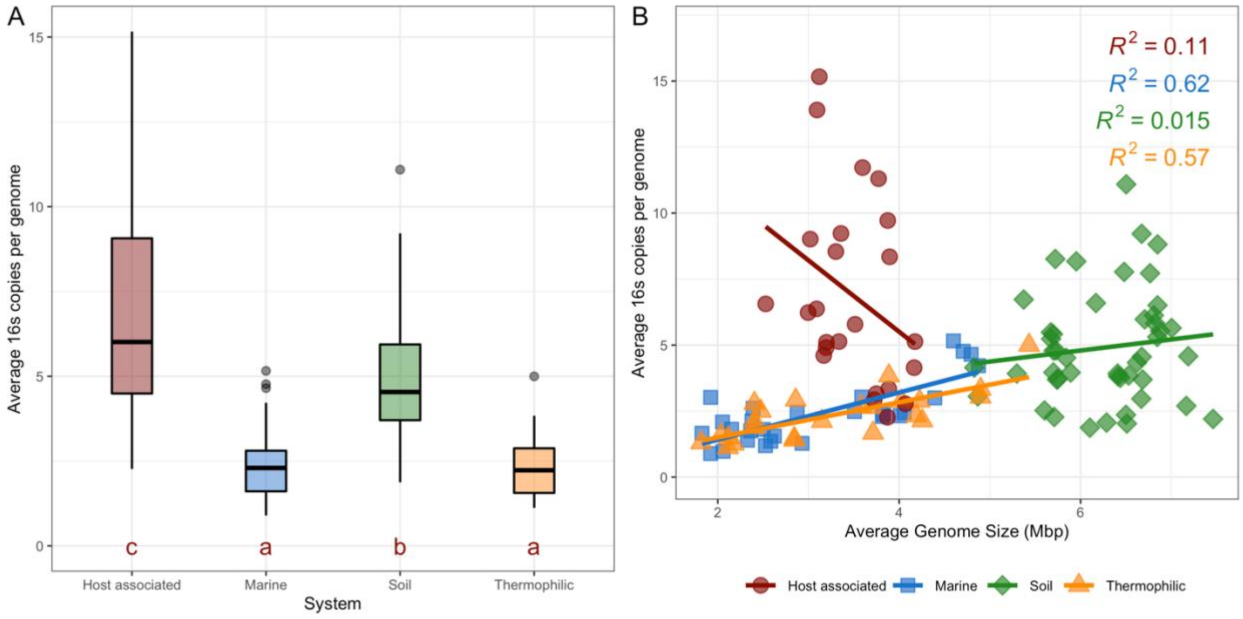

Supplemental Figure 4:

Here, and in the discussion, we discuss the relative abundance of each σ-factor as opposed to the more biologically relevant metric of average copies per genome. This is because the number of copies per genome relies on the calculation of genome size, thus resulting in autocorrelation in any analysis relating average genome size to average number of gene copies per genome. Still, we believe that the slopes for these relationships are biologically relevant and may be of interest to some readers. We therefore have provided a visualization for the average total number of σ-factors per genome (**A**), as well as the average number of specific σ-factor variants per genome (**B**).

A

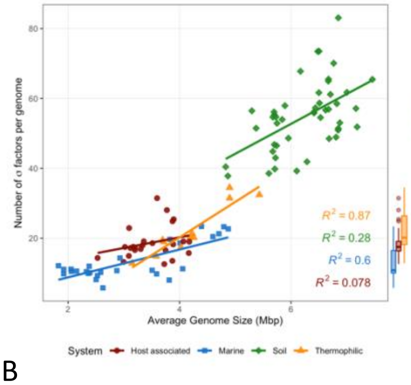
Number of σ factors per genome
Average Genome Size (Mbp)
R^2 = 0.87
R^2 = 0.28
R^2 = 0.6
R^2 = 0.078
System
Host associated
Marine
Soil
Thermophilic

B

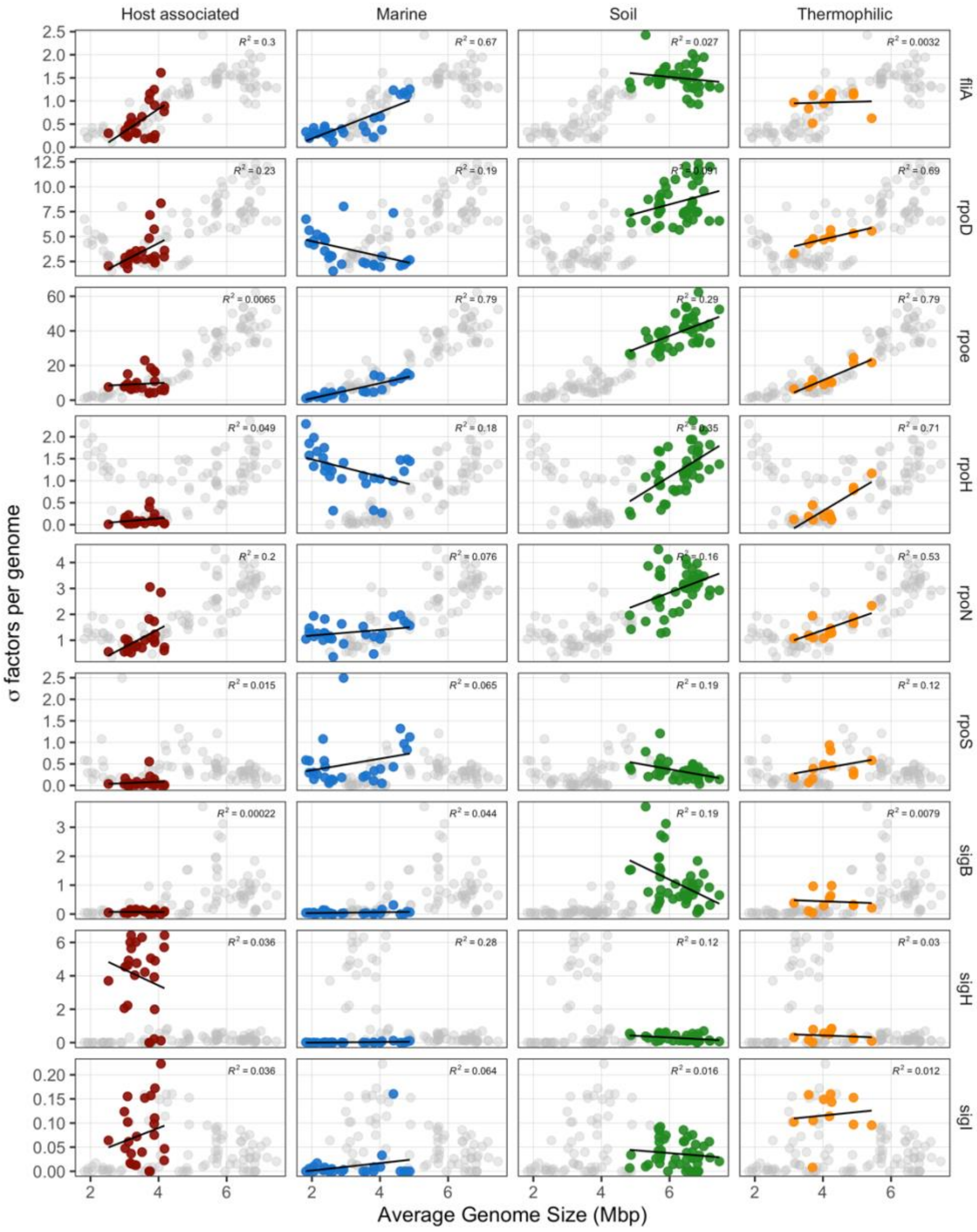
Host associated
Marine
Soil
Thermophilic
fliA
rpoD
rpoe
rpoH
rpoN
rpoS
sigB
sigH
sigI
σ factors per genome
Average Genome Size (Mbp)

Supplemental Figure 5:

Using a more stringent cutoff on the number of reads assigned to eukaryotes (<1%), the relationship between the number of reads which mapped to eukaryotes (% of total) and genome size **(**Mbp; **A)** and GC content **(**%; **B)**; as well as the relationship between genome size and GC content **(C).**

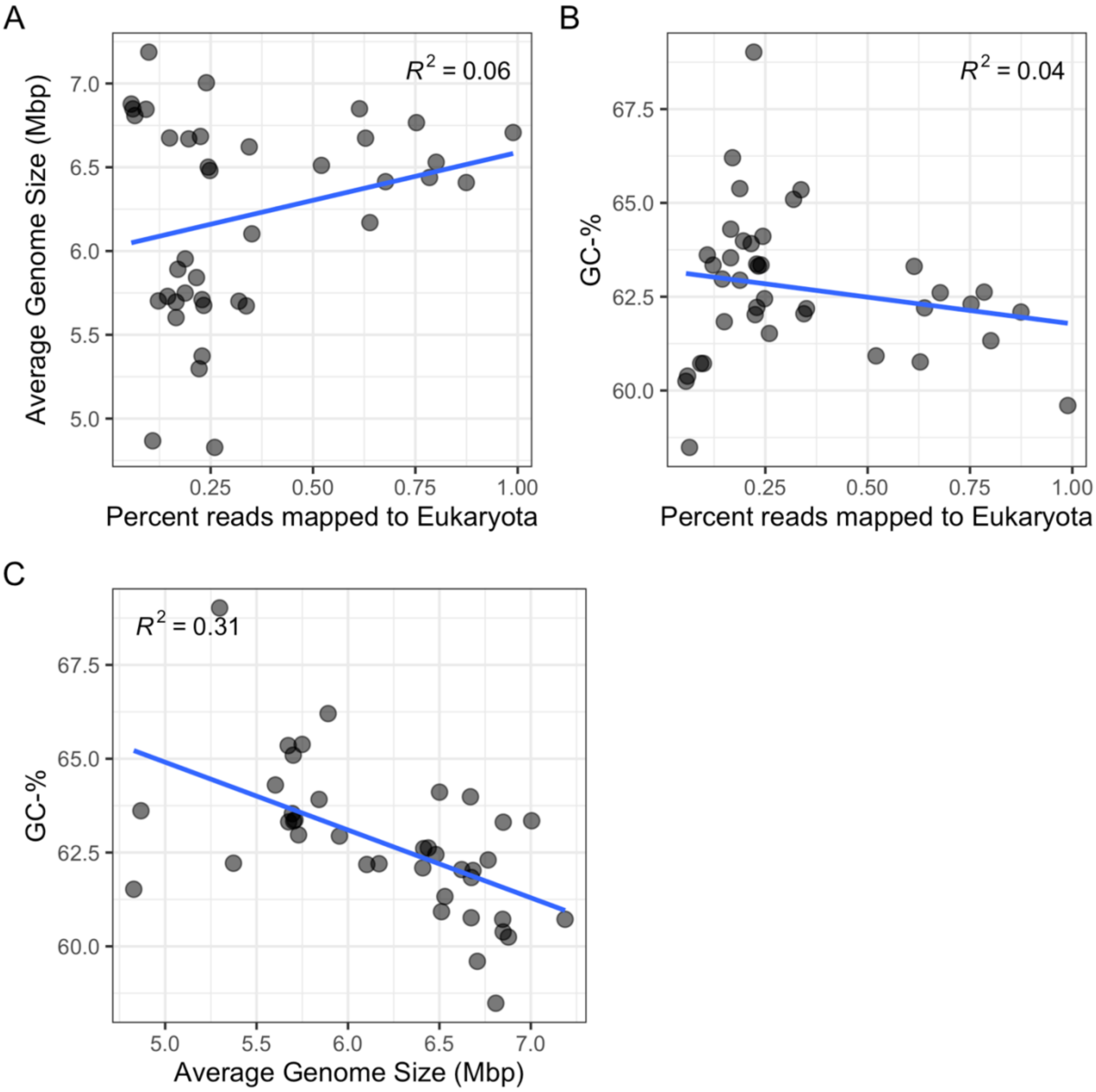

Supplemental Figure 6:

Relative abundance of σ^s gene *rpoS* as a function of average genome size, with source system indicated by color.

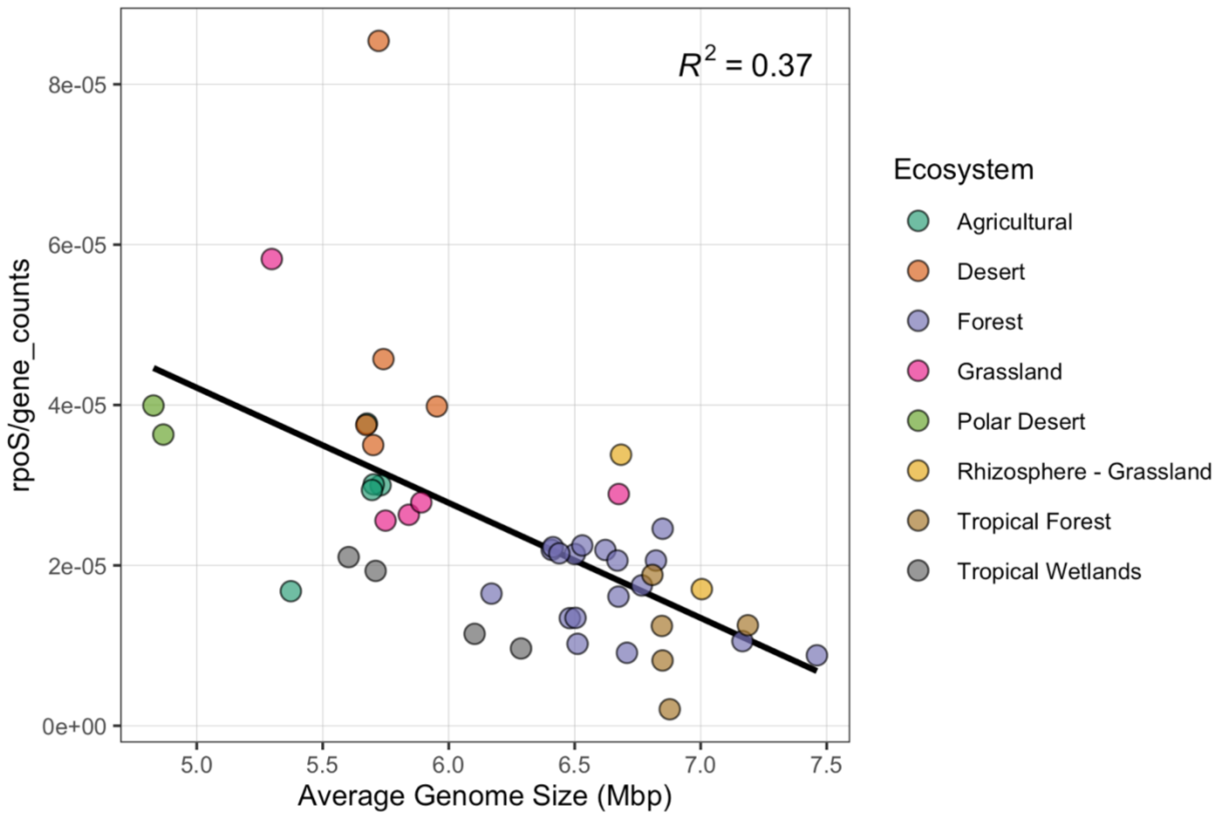

Supplement: xtab020_Supplemental_Files [file xtab020_supplemental_files.zip › Chuckran_2021_11_supp.pdf]
