# Supplementary material for: Variation in genomic traits of microbial communities among ecosystems
Source: FEMS Microbes. 2021 Dec 1;2:xtab020. doi: 10.1093/femsmc/xtab020 (PMC10117780; doi:10.1093/femsmc/xtab020)
Supplement: xtab020_Supplemental_Files [file xtab020_supplemental_files.zip › Chuckran_supplemental_refs.docx]

Supplemental References:

All unique publications associated with GOLD Study ID’s for isolates used in Figure 3. Genome size and GC content for selected isolates were gathered from the IMG/M database.

| GOLD Study ID | Is Published | Pubmed ID | Publication |
| --- | --- | --- | --- |
| Gs0032317 | Yes | 8590679 | (Kanzawa *et al.* 1995) |
| Gs0000008 | Yes | 9734056 | (Tamura, Hayakawa and Hatano 1998) |
| Gs0000005 | Yes | 10555311 | (Leonardo *et al.* 1999) |
| Gs0033830 | Yes | 12917641 | (Palenik *et al.* 2003) |
| Gs0019909 | Yes | 12917642 | (Rocap *et al.* 2003) |
| Gs0019904 | Yes | 12917642 | (Rocap *et al.* 2003) |
| Gs0011856 | Yes | 14742477 | (Vetriani *et al.* 2004) |
| Gs0034094 | Yes | 16452431 | (Beller *et al.* 2006) |
| Gs0012349 | Yes | 16707693 | (Mavromatis *et al.* 2006) |
| Gs0014564 | Yes | 16957257 | (Klotz *et al.* 2006) |
| Gs0016411 | Yes | 17030793 | (Makarova *et al.* 2006) |
| Gs0034100 | Yes | 17105352 | (Scott *et al.* 2006) |
| Gs0015350 | Yes | 17172329 | (Challacombe *et al.* 2007) |
| Gs0002033 | Yes | 17434157 | (Lapidus *et al.* 2008) |
| Gs0032479 | Yes | 17517606 | (Mwangi *et al.* 2007) |
| Gs0030913 | Yes | 17526795 | (Moran *et al.* 2007) |
| Gs0034652 | Yes | 17712418 | (Auerbach *et al.* 2007) |
| Gs0012009 | Yes | 17895995 | (Makarova *et al.* 2007) |
| Gs0034652 | Yes | 18060065 | (Smith *et al.* 2007) |
| Gs0019908 | Yes | 18159947 | (Kettler *et al.* 2007) |
| Gs0019910 | Yes | 18159947 | (Kettler *et al.* 2007) |
| Gs0012158 | Yes | 18263724 | (Anderson *et al.* 2008) |
| Gs0016411 | Yes | 18505588 | (Lee *et al.* 2008) |
| Gs0003140 | Yes | 18776029 | (Van De Werken *et al.* 2008) |
| Gs0016121 | Yes | 19000309 | (Podar *et al.* 2008) |
| Gs0002237 | Yes | 19033196 | (Sela *et al.* 2008) |
| Gs0019015 | Yes | 19136599 | (Reysenbach *et al.* 2009) |
| Gs0033955 | Yes | 19150844 | (Moran, McLaughlin and Sorek 2009) |
| Gs0000242 | Yes | 19270083 | (Barabote *et al.* 2009) |
| Gs0034075 | Yes | 19307556 | (Zhaxybayeva *et al.* 2009) |
| Gs0034652 | Yes | 19346311 | (Wattam *et al.* 2009) |
| Gs0001479 | Yes | 19429624 | (Setubal *et al.* 2009) |
| Gs0017186 | Yes | 19435847 | (Reno *et al.* 2009) |
| Gs0017421 | Yes | 19465526 | (Schübbe *et al.* 2009) |
| Gs0032623 | Yes | 19805210 | (Lauro *et al.* 2009) |
| Gs0011847 | Yes | 19948807 | (Young *et al.* 2010) |
| Gs0033967 | Yes | 20023027 | (Schmitz-Esser *et al.* 2010) |
| Gs0030907 | Yes | 20308297 | (Jeffrey *et al.* 2010) |
| Gs0016158 | Yes | 20428247 | (Woyke *et al.* 2010) |
| Gs0014532 | Yes | 20596068 | (Caro-Quintero *et al.* 2011) |
| Gs0032400 | Yes | 20596068 | (Caro-Quintero *et al.* 2011) |
| Gs0032403 | Yes | 20596068 | (Caro-Quintero *et al.* 2011) |
| Gs0034652 | Yes | 20601474 | (Chen *et al.* 2010) |
| Gs0019014 | Yes | 20601475 | (Tamas *et al.* 2010) |
| Gs0019015 | Yes | 20635162 | (Brumm *et al.* 2011) |
| Gs0003142 | Yes | 20851897 | (Elkins *et al.* 2010) |
| Gs0011852 | Yes | 20952571 | (Stein *et al.* 2010) |
| Gs0011862 | Yes | 21183664 | (Han *et al.* 2011) |
| Gs0000039 | Yes | 21216991 | (Blumer-Schuette *et al.* 2011) |
| Gs0000008 | Yes | 21304644 | (Mavrommatis *et al.* 2009) |
| Gs0000008 | Yes | 21304646 | (Ivanova *et al.* 2009) |
| Gs0012158 | Yes | 21304655 | (Anderson *et al.* 2009) |
| Gs0000008 | Yes | 21304705 | (Harmon-Smith *et al.* 2010) |
| Gs0012171 | Yes | 21304715 | (Giongo *et al.* 2010) |
| Gs0032479 | Yes | 21335390 | (Singer *et al.* 2011) |
| Gs0016615 | Yes | 21379339 | (Frese *et al.* 2011) |
| Gs0034356 | Yes | 21398537 | (van Passel *et al.* 2011a) |
| Gs0034356 | Yes | 21398538 | (van Passel *et al.* 2011b) |
| Gs0034356 | Yes | 21460084 | (Kant *et al.* 2011b) |
| Gs0034356 | Yes | 21460085 | (Kant *et al.* 2011a) |
| Gs0012170 | Yes | 21460088 | (Vishnivetskaya *et al.* 2011) |
| Gs0018994 | Yes | 21526192 | (Suen *et al.* 2011b) |
| Gs0012166 | Yes | 21622751 | (Liu *et al.* 2011) |
| Gs0011968 | Yes | 21677849 | (Kallimanis *et al.* 2011) |
| Gs0000008 | Yes | 21677853 | (Pukall *et al.* 2011) |
| Gs0000008 | Yes | 21677860 | (Land *et al.* 2011) |
| Gs0000060 | Yes | 21705587 | (Klippel *et al.* 2011) |
| Gs0004054 | Yes | 21714912 | (Tang *et al.* 2011) |
| Gs0018994 | Yes | 21914885 | (Suen *et al.* 2011a) |
| Gs0000102 | Yes | 22180806 | (Anderson *et al.* 2011b) |
| Gs0000102 | Yes | 22180810 | (Anderson *et al.* 2011a) |
| Gs0014528 | Yes | 22180812 | (Deangelis *et al.* 2011) |
| Gs0034308 | Yes | 22328742 | (Caro-Quintero *et al.* 2012) |
| Gs0014971 | Yes | 22493203 | (Davis *et al.* 2012) |
| Gs0000150 | Yes | 22529840 | (Techtmann *et al.* 2012) |
| Gs0014898 | Yes | 22536372 | (Martinez-Garcia *et al.* 2012) |
| Gs0011860 | Yes | 22675580 | (Chertkov *et al.* 2011) |
| Gs0000072 | Yes | 22675585 | (Kiss *et al.* 2011) |
| Gs0032306 | Yes | 22675599 | (Lucas-Elío *et al.* 2012a) |
| Gs0000249 | Yes | 22738439 | (Burdman and Walcott 2012) |
| Gs0034727 | Yes | 22768365 | (Klenk *et al.* 2012) |
| Gs0017185 | Yes | 22887658 | (Marx *et al.* 2012) |
| Gs0011834 | Yes | 23012283 | (Susanti *et al.* 2012) |
| Gs0019964 | Yes | 23045491 | (Lucas *et al.* 2012) |
| Gs0000093 | Yes | 23105050 | (Pester *et al.* 2012) |
| Gs0014887 | Yes | 23277585 | (Shih *et al.* 2013) |
| Gs0032305 | Yes | 23405355 | (Murugapiran *et al.* 2013) |
| Gs0019293 | Yes | 23408395 | (Mead *et al.* 2012) |
| Gs0015058 | Yes | 23450001 | (Neupane *et al.* 2012) |
| Gs0011853 | Yes | 23450099 | (Kappler *et al.* 2012) |
| Gs0034715 | Yes | 23450133 | (Rawat *et al.* 2012) |
| Gs0000117 | Yes | 23450211 | (Riedel *et al.* 2012) |
| Gs0032306 | Yes | 23458837 | (Lucas-Elío *et al.* 2012b) |
| Gs0000044 | Yes | 23516234 | (Aylward *et al.* 2013) |
| Gs0004704 | Yes | 23580711 | (Elkins *et al.* 2013) |
| Gs0034702 | Yes | 23814105 | (Khmelenina *et al.* 2013) |
| Gs0014971 | Yes | 23833133 | (Davis *et al.* 2013) |
| Gs0032341 | Yes | 23833140 | (Venkatramanan *et al.* 2013) |
| Gs0019860 | Yes | 23842652 | (Kamke *et al.* 2013) |
| Gs0014886 | Yes | 23851394 | (Rinke *et al.* 2013) |
| Gs0011854 | Yes | 24072863 | (Hirsch *et al.* 2013) |
| Gs0011862 | Yes | 24158554 | (Han *et al.* 2013) |
| Gs0015033 | Yes | 24286338 | (Zaremba-Niedzwiedzka *et al.* 2013) |
| Gs0014898 | Yes | 24451205 | (Thrash *et al.* 2014) |
| Gs0000117 | Yes | 24501645 | (Palaniappan *et al.* 2013) |
| Gs0034715 | Yes | 24501646 | (Rawat *et al.* 2013) |
| Gs0001313 | Yes | 24501649 | (Nakatsu *et al.* 2013) |
| Gs0000016 | Yes | 25197439 | (Reeve *et al.* 2014) |
| Gs0034309 | Yes | 25197485 | (Biddle *et al.* 2014) |
| Gs0030618 | Yes | 26044417 | (Hamilton *et al.* 2015) |
| Gs0016138 | Yes | 26231539 | (Smalley *et al.* 2015) |
| Gs0018994 | Yes | 26442136 | (Brumm, Land and Mead 2015) |
| Gs0019015 | Yes | 26500717 | (Brumm *et al.* 2015) |
| Gs0012172 | Yes | 26543115 | (Cadillo-Quiroz *et al.* 2015) |
| Gs0015051 | Yes | 26566423 | (Choi *et al.* 2015) |
| Gs0015003 | Yes | 27471578 | (Rice *et al.* 2016) |
| Gs0103574 | Yes | 27516514 | (Badhai, Whitman and Das 2016) |
| Gs0015003 | Yes | 28302769 | (Rice *et al.* 2017) |
| Gs0011834 | Yes | 28408663 | (Susanti *et al.* 2017) |
| Gs0015051 | Yes | 28491240 | (Laviad-Shitrit *et al.* 2017) |
| Gs0000008 | Yes | 28604660 | (Mukherjee *et al.* 2017) |
| Gs0000117 | Yes | 28604660 | (Mukherjee *et al.* 2017) |
| Gs0110196 | Yes | 28619793 | (Miroshnikov *et al.* 2017) |
| Gs0033970 | Yes | 29553575 | (Seshadri *et al.* 2018) |
| Gs0114533 | Yes | 29633519 | (Hesse *et al.* 2018) |
| Gs0114538 | Yes | 31596191 | (Bernard *et al.* 2020) |
| Gs0000699 | Yes | 10028265, 21118570 : (Guettler, Rumler and Jain 1999; McKinlay *et al.* 2010) | |
| Gs0015051 | Yes | 12054233, 28604660 : (Satomi *et al.* 2002; Mukherjee *et al.* 2017) | |
| Gs0015051 | Yes | 15653878, 28604660 : (Nedashkovskaya *et al.* 2005; Mukherjee *et al.* 2017) | |
| Gs0015051 | Yes | 17551043, 28604660 : (Goto *et al.* 2007; Mukherjee *et al.* 2017) | |
| Gs0015051 | Yes | 18676476, 28604660 : (Liu *et al.* 2008; Mukherjee *et al.* 2017) | |
| Gs0001092 | Yes | 19346307, 19465524 : (Kataeva *et al.* 2009; Yang *et al.* 2009) | |
| Gs0000008 | Yes | 21304653, 28604660 : (Copeland *et al.* 2009; Mukherjee *et al.* 2017) | |
| Gs0000008 | Yes | 21304663, 28604660 : (Pukall *et al.* 2009; Mukherjee *et al.* 2017) | |
| Gs0000008 | Yes | 21304704, 28604660 : (Pukall *et al.* 2010; Mukherjee *et al.* 2017) | |
| Gs0000008 | Yes | 21304717, 28604660 : (von Jan *et al.* 2010; Mukherjee *et al.* 2017) | |
| Gs0015051 | Yes | 26380636, 28604660 : (Sakamoto *et al.* 2015; Mukherjee *et al.* 2017) | |
| Gs0015051 | Yes | 26767091, 28604660 : (Aizenberg-Gershtein *et al.* 2016; Mukherjee *et al.* 2017) | |

REFERENCES:

Aizenberg-Gershtein Y, Izhaki I, Lapidus A *et al.* High quality permanent draft genome sequence of Phaseolibacter flectens ATCC 12775(T), a plant pathogen of French bean pods. *Stand Genomic Sci* 2016;**11**:4.

Anderson I, Risso C, Holmes D *et al.* Complete genome sequence of Ferroglobus placidus AEDII12DO. *Stand Genomic Sci* 2011a;**5**:50–60.

Anderson I, Rodriguez J, Susanti D *et al.* Genome sequence of Thermofilum pendens reveals an exceptional loss of biosynthetic pathways without genome reduction. *J Bacteriol* 2008;**190**:2957–65.

Anderson I, Wirth R, Lucas S *et al.* Complete genome sequence of Staphylothermus hellenicus P8 T. *Stand Genomic Sci* 2011b;**5**:12–20.

Anderson IJ, Sun H, Lapidus A *et al.* Complete genome sequence of staphylothermus marinus stetter and fiala 1986 type strain F1. *Stand Genomic Sci* 2009;**1**:183–8.

Auerbach RK, Tuanyok A, Probert WS *et al.* Yersinia pestis evolution on a small timescale: Comparison of whole genome sequences from North America. *PLoS One* 2007;**2**, DOI: 10.1371/journal.pone.0000770.

Aylward FO, Tremmel DM, Starrett GJ *et al.* Complete Genome of Serratia sp. Strain FGI 94, a Strain Associated with Leaf-Cutter Ant Fungus Gardens. *Genome Announc* 2013;**1**:e0023912.

Badhai J, Whitman WB, Das SK. Draft Genome Sequence of Chelatococcus sambhunathii Strain HT4T (DSM 18167T) Isolated from a Hot Spring in India. *Genome Announc* 2016;**4**, DOI: 10.1128/genomeA.00825-16.

Barabote RD, Xie G, Leu DH *et al.* Complete genome of the cellulolytic thermophile Acidothermus cellulolyticus IIB provides insights into its ecophysiological and evolutionary adaptations. *Genome Res* 2009;**19**:1033–42.

Beller HR, Chain PSG, Letain TE *et al.* The genome sequence of the obligately chemolithoautotrophic, facultatively anaerobic bacterium Thiobacillus denitrificans. *J Bacteriol* 2006;**188**:1473–88.

Bernard KA, Pacheco AL, Burdz T *et al.* Emendation of the Genus Auritidibacter Yassin et al. 2011 and Auritidibacter ignavus Yassin et al. 2011 based on features observed from Canadian and Swiss clinical isolates and whole-genome sequencing analysis. *Int J Syst Evol Microbiol* 2020;**70**:83–8.

Biddle AS, Leschine S, Huntemann M *et al.* The complete genome sequence of Clostridium indolis DSM 755(T.). *Stand Genomic Sci* 2014;**9**:1089–104.

Blumer-Schuette SE, Ozdemir I, Mistry D *et al.* Complete genome sequences for the anaerobic, extremely thermophilic plant biomass-degrading bacteria Caldicellulosiruptor hydrothermalis, Caldicellulosiruptor kristjanssonii, Caldicellulosiruptor kronotskyensis, Caldicellulosiruptor owensensis, and Caldicellulosiruptor lactoaceticus. *J Bacteriol* 2011;**193**:1483–4.

Brumm P, Hermanson S, Hochstein B *et al.* Mining Dictyoglomus turgidum for enzymatically active carbohydrases. *Appl Biochem Biotechnol* 2011;**163**:205–14.

Brumm P, Land ML, Hauser LJ *et al.* Complete genome sequences of Geobacillus sp. Y412MC52, a xylan-degrading strain isolated from obsidian hot spring in Yellowstone National Park. *Stand Genomic Sci* 2015;**10**:81.

Brumm PJ, Land ML, Mead DA. Complete genome sequence of Geobacillus thermoglucosidasius C56-YS93, a novel biomass degrader isolated from obsidian hot spring in Yellowstone National Park. *Stand Genomic Sci* 2015;**10**:73.

Burdman S, Walcott R. Acidovorax citrulli: Generating basic and applied knowledge to tackle a global threat to the cucurbit industry. *Mol Plant Pathol* 2012;**13**:805–15.

Cadillo-Quiroz H, Browne P, Kyrpides N *et al.* Complete Genome Sequence of Methanosphaerula palustris E1-9CT, a Hydrogenotrophic Methanogen Isolated from a Minerotrophic Fen Peatland. *Genome Announc* 2015;**3**, DOI: 10.1128/genomeA.01280-15.

Caro-Quintero A, Auchtung J, Deng J *et al.* Genome sequencing of five Shewanella baltica strains recovered from the oxic-anoxic interface of the baltic sea. *J Bacteriol* 2012;**194**:1236–1236.

Caro-Quintero A, Deng J, Auchtung J *et al.* Unprecedented levels of horizontal gene transfer among spatially co-occurring Shewanella bacteria from the Baltic Sea. *ISME J* 2011;**5**:131–40.

Challacombe JF, Duncan AJ, Brettin TS *et al.* Complete genome sequence of Haemophilus somnus (Histophilus somni) strain 129Pt and comparison to Haemophilus ducreyi 35000HP and Haemophilus influenzae Rd. *J Bacteriol* 2007;**189**:1890–8.

Chen J, Xie G, Han S *et al.* Whole genome sequences of two Xylella fastidiosa strains (M12 and M23) causing almond leaf scorch disease in California. *J Bacteriol* 2010;**192**:4534.

Chertkov O, Brown PJ, Kysela DT *et al.* Complete genome sequence of Hirschia baltica type strain (IFAM 1418 T). *Stand Genomic Sci* 2011;**5**:287–97.

Choi DH, Ahn C, Jang G Il *et al.* High-quality draft genome sequence of Gracilimonas tropica CL-CB462T (DSM 19535T), isolated from a Synechococcus culture. *Stand Genomic Sci* 2015;**10**, DOI: 10.1186/s40793-015-0088-8.

Copeland A, Sikorski J, Lapidus A *et al.* Complete genome sequence of Atopobium parvulum type strain (IPP 1246). *Stand Genomic Sci* 2009;**1**:166–73.

Davis JR, Goodwin L, Teshima H *et al.* Genome sequence of Streptomyces viridosporus strain T7A ATCC 39115, a lignin-degrading actinomycete. *Genome Announc* 2013;**1**, DOI: 10.1128/genomeA.00416-13.

Davis JR, Goodwin LA, Woyke T *et al.* Genome sequence of Amycolatopsis sp. strain ATCC 39116, a plant biomass-degrading actinomycete. *J Bacteriol* 2012;**194**:2396–7.

Deangelis KM, D’Haeseleer P, Chivian D *et al.* Complete genome sequence of “Enterobacter lignolyticus” SCF1. *Stand Genomic Sci* 2011;**5**:69–85.

Elkins JG, Hamilton-Brehm SD, Lucas S *et al.* Complete Genome Sequence of the Hyperthermophilic Sulfate-Reducing Bacterium Thermodesulfobacterium geofontis OPF15T. *Genome Announc* 2013;**1**:e0016213.

Elkins JG, Lochner A, Hamilton-Brehm SD *et al.* Complete genome sequence of the cellulolytic thermophile Caldicellulosiruptor obsidiansis OB47T. *J Bacteriol* 2010;**192**:6099–100.

Frese SA, Benson AK, Tannock GW *et al.* The evolution of host specialization in the vertebrate gut symbiont Lactobacillus reuteri. *PLoS Genet* 2011;**7**, DOI: 10.1371/journal.pgen.1001314.

Giongo A, Tyler HL, Zipperer UN *et al.* Two genome sequences of the same bacterial strain, Gluconacetobacter diazotrophicus PAl 5, suggest a new standard in genome sequence submission. *Stand Genomic Sci* 2010;**2**:309–17.

Goto K, Mochida K, Kato Y *et al.* Proposal of six species of moderately thermophilic, acidophilic, endospore-forming bacteria: Alicyclobacillus contaminans sp. nov., Alicyclobacillus fastidiosus sp. nov., Alicyclobacillus kakegawensis sp. nov., Alicyclobacillus macrosporangiidus sp. nov.,. *Int J Syst Evol Microbiol* 2007;**57**:1276–85.

Guettler M V, Rumler D, Jain MK. Actinobacillus succinogenes sp. nov., a novel succinic-acid-producing strain from the bovine rumen. *Int J Syst Bacteriol* 1999;**49 Pt 1**:207–16.

Hamilton R, Kits KD, Ramonovskaya VA *et al.* Draft genomes of gammaproteobacterial methanotrophs isolated from terrestrial ecosystems. *Genome Announc* 2015;**3**, DOI: 10.1128/genomeA.00515-15.

Han J-I, Spain JC, Leadbetter JR *et al.* Genome of the Root-Associated Plant Growth-Promoting Bacterium Variovorax paradoxus Strain EPS. *Genome Announc* 2013;**1**, DOI: 10.1128/genomeA.00843-13.

Han JI, Choi HK, Lee SW *et al.* Complete genome sequence of the metabolically versatile plant growth-promoting endophyte Variovorax paradoxus S110. *J Bacteriol* 2011;**193**:1183–90.

Harmon-Smith M, Celia L, Chertkov O *et al.* Complete genome sequence of Sebaldella termitidis type strain (NCTC 11300 T). *Stand Genomic Sci* 2010;**2**:220–7.

Hesse C, Schulz F, Bull CT *et al.* Genome-based evolutionary history of Pseudomonas spp. *Environ Microbiol* 2018;**20**:2142–59.

Hirsch AM, Alvarado J, Bruce D *et al.* Complete Genome Sequence of Micromonospora Strain L5, a Potential Plant-Growth-Regulating Actinomycete, Originally Isolated from Casuarina equisetifolia Root Nodules. *Genome Announc* 2013;**1**, DOI: 10.1128/genomeA.00759-13.

Ivanova N, Sikorski J, Sims D *et al.* Complete genome sequence of Sanguibacter keddieii type strain (ST-74 T). *Stand Genomic Sci* 2009;**1**:110–8.

von Jan M, Lapidus A, Del Rio TG *et al.* Complete genome sequence of Archaeoglobus profundus type strain (AV18). *Stand Genomic Sci* 2010;**2**:327–46.

Jeffrey BM, Suchland RJ, Quinn KL *et al.* Genome sequencing of recent clinical Chlamydia trachomatis strains identifies loci associated with tissue tropism and regions of apparent recombination. *Infect Immun* 2010;**78**:2544–53.

Kallimanis A, Labutti KM, Lapidus A *et al.* Complete genome sequence of Arthrobacter phenanthrenivorans type strain (Sphe3). *Stand Genomic Sci* 2011;**4**:123–30.

Kamke J, Sczyrba A, Ivanova N *et al.* Single-cell genomics reveals complex carbohydrate degradation patterns in poribacterial symbionts of marine sponges. *ISME J* 2013;**7**:2287–300.

Kant R, van Passe MWJ, Palva A *et al.* Genome sequence of Chthoniobacter flavus Ellin428, an aerobic heterotrophic soil bacterium. *J Bacteriol* 2011a;**193**:2902–3.

Kant R, van Passel MWJ, Sangwan P *et al.* Genome sequence of “Pedosphaera parvula” Ellin514, an aerobic verrucomicrobial isolate from pasture soil. *J Bacteriol* 2011b;**193**:2900–1.

Kanzawa Y, Harada A, Takeuchi M *et al.* Bacillus curdlanolyticus sp. nov. and Bacillus kobensis sp. nov., which hydrolyze resistant curdlan. *Int J Syst Bacteriol* 1995;**45**:515–21.

Kappler U, Davenport K, Beatson S *et al.* Complete genome sequence of the facultatively chemolithoautotrophic and methylotrophic alpha Proteobacterium Starkeya novella type strain (ATCC 8093T). *Stand Genomic Sci* 2012;**7**:44–58.

Kataeva IA, Yang S-J, Dam P *et al.* Genome sequence of the anaerobic, thermophilic, and cellulolytic bacterium “Anaerocellum thermophilum” DSM 6725. *J Bacteriol* 2009;**191**:3760–1.

Kettler GC, Martiny AC, Huang K *et al.* Patterns and implications of gene gain and loss in the evolution of Prochlorococcus. *PLoS Genet* 2007;**3**:2515–28.

Khmelenina VN, Beck DAC, Munk C *et al.* Draft Genome Sequence of Methylomicrobium buryatense Strain 5G, a Haloalkaline-Tolerant Methanotrophic Bacterium. *Genome Announc* 2013;**1**, DOI: 10.1128/genomeA.00053-13.

Kiss H, Nett M, Domin N *et al.* Complete genome sequence of the filamentous gliding predatory bacterium Herpetosiphon aurantiacus type strain (114-95T). *Stand Genomic Sci* 2011;**5**:356–70.

Klenk H-P, Held B, Lucas S *et al.* Genome sequence of the soil bacterium Saccharomonospora azurea type strain (NA-128(T)). *Stand Genomic Sci* 2012;**6**:220–9.

Klippel B, Lochner A, Bruce DC *et al.* Complete genome sequence of the marine cellulose- and xylan-degrading bacterium Glaciecola sp. strain 4H-3-7+YE-5. *J Bacteriol* 2011;**193**:4547–8.

Klotz MG, Arp DJ, Chain PSG *et al.* Complete genome sequence of the marine, chemolithoautotrophic, ammonia-oxidizing bacterium Nitrosococcus oceani ATCC 19707. *Appl Environ Microbiol* 2006;**72**:6299–315.

Land M, Held B, Gronow S *et al.* Non-contiguous finished genome sequence of Bacteroides coprosuis type strain (PC139 T). *Stand Genomic Sci* 2011;**4**:233–43.

Lapidus A, Goltsman E, Auger S *et al.* Extending the Bacillus cereus group genomics to putative food-borne pathogens of different toxicity. *Chem Biol Interact* 2008;**171**:236–49.

Lauro FM, McDougald D, Thomas T *et al.* The genomic basis of trophic strategy in marine bacteria. *Proc Natl Acad Sci U S A* 2009;**106**:15527–33.

Laviad-Shitrit S, Göker M, Huntemann M *et al.* High quality permanent draft genome sequence of Chryseobacterium bovis DSM 19482T, isolated from raw cow milk. *Stand Genomic Sci* 2017;**12**:31.

Lee JH, Karamychev VN, Kozyavkin SA *et al.* Comparative genomic analysis of the gut bacterium Bifidobacterium longum reveals loci susceptible to deletion during pure culture growth. *BMC Genomics* 2008;**9**, DOI: 10.1186/1471-2164-9-247.

Leonardo MR, Moser DP, Barbieri E *et al.* Shewanella pealeana sp. nov., a member of the microbial community associated with the accessory nidamental gland of the squid Loligo pealei. *Int J Syst Bacteriol* 1999;**49**:1341–51.

Liu C, Finegold SM, Song Y *et al.* Reclassification of Clostridium coccoides, Ruminococcus hansenii, Ruminococcus hydrogenotrophicus, Ruminococcus luti, Ruminococcus productus and Ruminococcus schinkii as Blautia coccoides gen. nov., comb. nov., Blautia hansenii comb. nov., Blautia hydroge. *Int J Syst Evol Microbiol* 2008;**58**:1896–902.

Liu S, Leathers TD, Copeland A *et al.* Complete genome sequence of Lactobacillus buchneri NRRL B-30929, a novel strain from a commercial ethanol plant. *J Bacteriol* 2011;**193**:4019–20.

Lucas-Elío P, Goodwin L, Woyke T *et al.* Complete genome sequence of the melanogenic marine bacterium Marinomonas mediterranea type strain (MMB-1 T). *Stand Genomic Sci* 2012a;**6**:63–73.

Lucas-Elío P, Goodwin L, Woyke T *et al.* Complete genome sequence of Marinomonas posidonica type strain (IVIA-Po-181(T)). *Stand Genomic Sci* 2012b;**7**:31–43.

Lucas S, Han J, Lapidus A *et al.* Complete genome sequence of the thermophilic, piezophilic, heterotrophic bacterium Marinitoga piezophila KA3. *J Bacteriol* 2012;**194**:5974–5.

Makarova K, Slesarev A, Wolf Y *et al.* Comparative genomics of the lactic acid bacteria. *Proc Natl Acad Sci U S A* 2006;**103**:15611–6.

Makarova KS, Omelchenko M V., Gaidamakova EK *et al.* Deinococcus geothermalis: The pool of extreme radiation resistance genes shrinks. *PLoS One* 2007;**2**, DOI: 10.1371/journal.pone.0000955.

Martinez-Garcia M, Brazel DM, Swan BK *et al.* Capturing single cell genomes of active polysaccharide degraders: An unexpected contribution of verrucomicrobia. *PLoS One* 2012;**7**, DOI: 10.1371/journal.pone.0035314.

Marx CJ, Bringel F, Chistoserdova L *et al.* Complete genome sequences of six strains of the genus Methylobacterium. *J Bacteriol* 2012;**194**:4746–8.

Mavromatis K, Doyle CK, Lykidis A *et al.* The genome of the obligately intracellular bacterium Ehrlichia canis reveals themes of complex membrane structure and immune evasion strategies. *J Bacteriol* 2006;**188**:4015–23.

Mavrommatis K, Pukall R, Rohde C *et al.* Complete genome sequence of Cryptobacterium curtum type strain (12-3 T). *Stand Genomic Sci* 2009;**1**:93–100.

McKinlay JB, Laivenieks M, Schindler BD *et al.* A genomic perspective on the potential of Actinobacillus succinogenes for industrial succinate production. *BMC Genomics* 2010;**11**:680.

Mead DA, Lucas S, Copeland A *et al.* Complete genome sequence of Paenibacillus strain Y4.12MC10, a novel Paenibacillus lautus strain isolated from obsidian hot spring in yellowstone national park. *Stand Genomic Sci* 2012;**6**:366–85.

Miroshnikov KK, Didriksen A, Naumoff DG *et al.* Draft Genome Sequence of Methylocapsa palsarum NE2T, an Obligate Methanotroph from Subarctic Soil. *Genome Announc* 2017;**5**, DOI: 10.1128/genomeA.00504-17.

Moran MA, Belas R, Schell MA *et al.* Ecological genomics of marine roseobacters. *Appl Environ Microbiol* 2007;**73**:4559–69.

Moran NA, McLaughlin HJ, Sorek R. The dynamics and time scale of ongoing genomic erosion in symbiotic bacteria. *Science (80- )* 2009;**323**:379–82.

Mukherjee S, Seshadri R, Varghese NJ *et al.* 1,003 reference genomes of bacterial and archaeal isolates expand coverage of the tree of life. *Nat Biotechnol* 2017;**35**:676–83.

Murugapiran SK, Huntemann M, Wei C-L *et al.* Whole Genome Sequencing of Thermus oshimai JL-2 and Thermus thermophilus JL-18, Incomplete Denitrifiers from the United States Great Basin. *Genome Announc* 2013;**1**, DOI: 10.1128/genomeA.00106-12.

Mwangi MM, Shang WW, Zhou Y *et al.* Tracking the in vivo evolution of multidrug resistance in Staphylococcus aureus by whole-genome sequencing. *Proc Natl Acad Sci U S A* 2007;**104**:9451–6.

Nakatsu CH, Barabote R, Thompson S *et al.* Complete genome sequence of Arthrobacter sp. strain FB24. *Stand Genomic Sci* 2013;**9**:106–16.

Nedashkovskaya OI, Kim SB, Lysenko AM *et al.* Description of Aquimarina muelleri gen. nov., sp. nov., and proposal of the reclassification of [Cytophaga] latercula Lewin 1969 as Stanierella latercula gen. nov., comb. nov. *Int J Syst Evol Microbiol* 2005;**55**:225–9.

Neupane S, Finlay RD, Kyrpides NC *et al.* Complete genome sequence of the plant-associated Serratia plymuthica strain AS13. *Stand Genomic Sci* 2012;**7**:22–30.

Palaniappan K, Meier-Kolthoff JP, Teshima H *et al.* Genome sequence of the moderately thermophilic sulfur-reducing bacterium Thermanaerovibrio velox type strain (Z-9701(T)) and emended description of the genus Thermanaerovibrio. *Stand Genomic Sci* 2013;**9**:57–70.

Palenik B, Brahamsha B, Larimer FW *et al.* The genome of a motile marine Synechococcus. *Nature* 2003;**424**:1037–42.

van Passel MWJ, Kant R, Palva A *et al.* Genome sequence of Victivallis vadensis ATCC BAA-548, an anaerobic bacterium from the phylum Lentisphaerae, isolated from the human gastrointestinal tract. *J Bacteriol* 2011a;**193**:2373–4.

van Passel MWJ, Kant R, Palva A *et al.* Genome sequence of the Verrucomicrobium Opitutus terrae PB90-1, an abundant inhabitant of rice paddy soil ecosystems. *J Bacteriol* 2011b;**193**:2367–8.

Pester M, Brambilla E, Alazard D *et al.* Complete genome sequences of Desulfosporosinus orientis DSM765T, Desulfosporosinus youngiae DSM17734T, Desulfosporosinus meridiei DSM13257T, and Desulfosporosinus acidiphilus DSM22704T. *J Bacteriol* 2012;**194**:6300–1.

Podar M, Anderson I, Makarova KS *et al.* A genomic analysis of the archaeal system Ignicoccus hospitalis-Nanoarchaeum equitans. *Genome Biol* 2008;**9**, DOI: 10.1186/gb-2008-9-11-r158.

Pukall R, Lapidus A, Glavina Del Rio T *et al.* Complete genome sequence of Conexibacter woesei type strain (ID131577 T). *Stand Genomic Sci* 2010;**2**:212–9.

Pukall R, Lapidus A, Nolan M *et al.* Complete genome sequence of Slackia heliotrinireducens type strain (RHS 1). *Stand Genomic Sci* 2009;**1**:234–41.

Pukall R, Zeytun A, Lucas S *et al.* Complete genome sequence of Deinococcus maricopensis type strain (LB-34 T). *Stand Genomic Sci* 2011;**4**:163–72.

Rawat SR, Männistö MK, Starovoytov V *et al.* Complete genome sequence of Terriglobus saanensis type strain SP1PR4(T), an Acidobacteria from tundra soil. *Stand Genomic Sci* 2012;**7**:59–69.

Rawat SR, Männistö MK, Starovoytov V *et al.* Complete genome sequence of Granulicella mallensis type strain MP5ACTX8(T), an acidobacterium from tundra soil. *Stand Genomic Sci* 2013;**9**:71–82.

Reeve W, Ardley J, Tian R *et al.* Genome sequence of the Listia angolensis microsymbiont Microvirga lotononidis strain WSM3557(T.). *Stand Genomic Sci* 2014;**9**:540–50.

Reno ML, Held NL, Fields CJ *et al.* Biogeography of the Sulfolobus islandicus pan-genome. *Proc Natl Acad Sci U S A* 2009;**106**:8605–10.

Reysenbach AL, Hamamura N, Podar M *et al.* Complete and draft genome sequences of six members of the aquificales. *J Bacteriol* 2009;**191**:1992–3.

Rice MC, Norton JM, Stein LY *et al.* Complete Genome Sequence of Nitrosomonas cryotolerans ATCC 49181, a Phylogenetically Distinct Ammonia-Oxidizing Bacterium Isolated from Arctic Waters. *Genome Announc* 2017;**5**, DOI: 10.1128/genomeA.00011-17.

Rice MC, Norton JM, Valois F *et al.* Complete genome of Nitrosospira briensis C-128, an ammonia-oxidizing bacterium from agricultural soil. *Stand Genomic Sci* 2016;**11**:46.

Riedel T, Held B, Nolan M *et al.* Genome sequence of the orange-pigmented seawater bacterium Owenweeksia hongkongensis type strain (UST20020801T). *Stand Genomic Sci* 2012;**7**:120–30.

Rinke C, Schwientek P, Sczyrba A *et al.* Insights into the phylogeny and coding potential of microbial dark matter. *Nature* 2013;**499**:431–7.

Rocap G, Larimer FW, Lamerdin J *et al.* Genome divergence in two Prochlorococcus ecotypes reflects oceanic niche differentiation. *Nature* 2003;**424**:1042–7.

Sakamoto M, Lapidus AL, Han J *et al.* High quality draft genome sequence of Bacteroides barnesiae type strain BL2(T) (DSM 18169(T)) from chicken caecum. *Stand Genomic Sci* 2015;**10**:48.

Satomi M, Kimura B, Hamada T *et al.* Phylogenetic study of the genus Oceanospirillum based on 16S rRNA and gyrB genes: emended description of the genus Oceanospirillum, description of Pseudospirillum gen. nov., Oceanobacter gen. nov. and Terasakiella gen. nov. and transfer of Oceanospirillum. *Int J Syst Evol Microbiol* 2002;**52**:739–47.

Schmitz-Esser S, Tischler P, Arnold R *et al.* The genome of the amoeba symbiont “Candidatus Amoebophilus asiaticus” reveals common mechanisms for host cell interaction among amoeba-associated bacteria. *J Bacteriol* 2010;**192**:1045–57.

Schübbe S, Williams TJ, Xie G *et al.* Complete genome sequence of the chemolithoautotrophic marine magnetotactic coccus strain MC-1. *Appl Environ Microbiol* 2009;**75**:4835–52.

Scott KM, Sievert SM, Abril FN *et al.* The genome of deep-sea vent chemolithoautotroph Thiomicrospira crunogena XCL-2. *PLoS Biol* 2006;**4**:2196–212.

Sela DA, Chapman J, Adeuya A *et al.* The genome sequence of Bifidobacterium longum subsp. infantis reveals adaptations for milk utilization within the infant microbiome. *Proc Natl Acad Sci U S A* 2008;**105**:18964–9.

Seshadri R, Leahy SC, Attwood GT *et al.* Cultivation and sequencing of rumen microbiome members from the Hungate1000 Collection. *Nat Biotechnol* 2018;**36**:359–67.

Setubal JC, Dos Santos P, Goldman BS *et al.* Genome sequence of Azotobacter vinelandii, an obligate aerobe specialized to support diverse anaerobic metabolic processes. *J Bacteriol* 2009;**191**:4534–45.

Shih PM, Wu D, Latifi A *et al.* Improving the coverage of the cyanobacterial phylum using diversity-driven genome sequencing. *Proc Natl Acad Sci U S A* 2013;**110**:1053–8.

Singer E, Webb EA, Nelson WC *et al.* Genomic potential of Marinobacter aquaeolei, a biogeochemical “Opportunitroph.” *Appl Environ Microbiol* 2011;**77**:2763–71.

Smalley NE, Taipale S, De Marco P *et al.* Functional and genomic diversity of methylotrophic Rhodocyclaceae: description of Methyloversatilis discipulorum sp. nov. *Int J Syst Evol Microbiol* 2015;**65**:2227–33.

Smith TJ, Hill KK, Foley BT *et al.* Analysis of the neurotoxin complex genes in Clostridium botulinum A1-A4 and B1 strains: BoNT/A3, /Ba4 and /B1 clusters are located within plasmids. *PLoS One* 2007;**2**, DOI: 10.1371/journal.pone.0001271.

Stein LY, Yoon S, Semrau JD *et al.* Genome sequence of the obligate methanotroph Methylosinus trichosporium strain OB3b. *J Bacteriol* 2010;**192**:6497–8.

Suen G, Stevenson DM, Bruce DC *et al.* Complete genome of the cellulolytic ruminal bacterium Ruminococcus albus 7. *J Bacteriol* 2011a;**193**:5574–5.

Suen G, Weimer PJ, Stevenson DM *et al.* The complete genome sequence of fibrobacter succinogenes s85 reveals a cellulolytic and metabolic specialist. *PLoS One* 2011b;**6**, DOI: 10.1371/journal.pone.0018814.

Susanti D, Johnson EF, Lapidus A *et al.* Permanent Draft Genome Sequence of Desulfurococcus amylolyticus Strain Z-533T, a Peptide and Starch Degrader Isolated from Thermal Springs in the Kamchatka Peninsula and Kunashir Island, Russia. *Genome Announc* 2017;**5**, DOI: 10.1128/genomeA.00078-17.

Susanti D, Johnson EF, Rodriguez JR *et al.* Complete genome sequence of Desulfurococcus fermentans, a hyperthermophilic cellulolytic crenarchaeon isolated from a freshwater hot spring in Kamchatka, Russia. *J Bacteriol* 2012;**194**:5703–4.

Tamas I, Dedysh SN, Liesack W *et al.* Complete genome sequence of Beijerinckia indica subsp. indica. *J Bacteriol* 2010;**192**:4532–3.

Tamura T, Hayakawa M, Hatano K. A new genus of the order Actinomycetales, Cryptosporangium gen. nov., with descriptions of Cryptosporangium arvum sp. nov. and Cryptosporangium japonicum sp. nov. *Int J Syst Bacteriol* 1998;**48 Pt 3**:995–1005.

Tang KH, Barry K, Chertkov O *et al.* Complete genome sequence of the filamentous anoxygenic phototrophic bacterium Chloroflexus aurantiacus. *BMC Genomics* 2011;**12**, DOI: 10.1186/1471-2164-12-334.

Techtmann SM, Lebedinsky A V., Colman AS *et al.* Evidence for horizontal gene transfer of anaerobic carbon monoxide dehydrogenases. *Front Microbiol* 2012;**3**, DOI: 10.3389/fmicb.2012.00132.

Thrash JC, Temperton B, Swan BK *et al.* Single-cell enabled comparative genomics of a deep ocean SAR11 bathytype. *ISME J* 2014;**8**:1440–51.

Venkatramanan R, Prakash O, Woyke T *et al.* Genome sequences for three denitrifying bacterial strains isolated from a uranium- and nitrate-contaminated subsurface environment. *Genome Announc* 2013;**1**, DOI: 10.1128/genomeA.00449-13.

Vetriani C, Speck MD, Ellor S V. *et al.* Thermovibrio ammonificans sp. nov., a thermophilic, chemolithotrophic, nitrate-ammonifying bacterium from deep-sea hydrothermal vents. *Int J Syst Evol Microbiol* 2004;**54**:175–81.

Vishnivetskaya TA, Lucas S, Copeland A *et al.* Complete genome sequence of the thermophilic bacterium Exiguobacterium sp. AT1b. *J Bacteriol* 2011;**193**:2880–1.

Wattam AR, Williams KP, Snyder EE *et al.* Analysis of ten Brucella genomes reveals evidence for horizontal gene transfer despite a preferred intracellular lifestyle. *J Bacteriol* 2009;**191**:3569–79.

Van De Werken HJG, Verhaart MRA, VanFossen AL *et al.* Hydrogenomics of the extremely thermophilic bacterium Caldicellulosiruptor saccharolyticus. *Appl Environ Microbiol* 2008;**74**:6720–9.

Woyke T, Tighe D, Mavromatis K *et al.* One bacterial cell, one complete genome. *PLoS One* 2010;**5**, DOI: 10.1371/journal.pone.0010314.

Yang S-J, Kataeva I, Hamilton-Brehm SD *et al.* Efficient degradation of lignocellulosic plant biomass, without pretreatment, by the thermophilic anaerobe “Anaerocellum thermophilum” DSM 6725. *Appl Environ Microbiol* 2009;**75**:4762–9.

Young M, Artsatbanov V, Beller HR *et al.* Genome sequence of the fleming strain of Micrococcus luteus, a simple free-living actinobacterium. *J Bacteriol* 2010;**192**:841–60.

Zaremba-Niedzwiedzka K, Viklund J, Zhao W *et al.* Single-cell genomics reveal low recombination frequencies in freshwater bacteria of the SAR11 clade. *Genome Biol* 2013;**14**, DOI: 10.1186/gb-2013-14-11-r130.

Zhaxybayeva O, Swithers KS, Lapierre P *et al.* On the chimeric nature, thermophilic origin, and phylogenetic placement of the Thermotogales. *Proc Natl Acad Sci U S A* 2009;**106**:5865–70.
